# Robotic Manipulation and Capture in Space: A Survey

**DOI:** 10.3389/frobt.2021.686723

**Published:** 2021-07-19

**Authors:** Evangelos Papadopoulos, Farhad Aghili, Ou Ma, Roberto Lampariello

**Affiliations:** ^1^ Control Systems Lab, School of Mechanical Engineering, National Technical University of Athens, Athens, Greece; ^2^ Space Exploration, Canadian Space Agency (CSA), Montreal, QC, Canada; ^3^ Intelligent Robotics and Autonomous Systems Lab, College of Engineering and Applied Science, University of Cincinnati, Cincinnati, OH, United States; ^4^ Institute of Robotics and Mechatronics, German Aerospace Center (DLR), Oberpfaffenhofen, Germany

**Keywords:** space robotics, on-orbit servicing, robotic capture on orbit, manipulation in orbit, ground testing of space robots, dynamics and control of space robots, contact dynamics, grippers for space

## Abstract

Space exploration and exploitation depend on the development of on-orbit robotic capabilities for tasks such as servicing of satellites, removing of orbital debris, or construction and maintenance of orbital assets. Manipulation and capture of objects on-orbit are key enablers for these capabilities. This survey addresses fundamental aspects of manipulation and capture, such as the dynamics of space manipulator systems (SMS), i.e., satellites equipped with manipulators, the contact dynamics between manipulator grippers/payloads and targets, and the methods for identifying properties of SMSs and their targets. Also, it presents recent work of sensing pose and system states, of motion planning for capturing a target, and of feedback control methods for SMS during motion or interaction tasks. Finally, the paper reviews major ground testing testbeds for capture operations, and several notable missions and technologies developed for capture of targets on-orbit.

## Introduction

Space exploration and exploitation depend on tasks such as inspecting, refueling, upgrading, repairing, or rescuing satellites, removing of orbital debris, and construction and maintenance of large orbital assets and infrastructures. Until now, all notable servicing tasks have been performed at Low Earth Orbit (LEO) by astronaut Extravehicular Activities (EVAs). However, EVAs are by nature risky operations requiring careful planning and preparation. Unfortunately, this increases mission costs and turn-around time drastically, making servicing missions too costly, of prolonged development, or even unfeasible. For critical space assets located in Geosynchronous Orbits (GEO) or other high-altitude orbits, EVA is not even an option in the foreseeable future.

To execute on-orbit tasks being inaccessible to, or too dangerous for humans, robotic on-orbit servicing (OOS) can be employed, with tasks to be performed by *space manipulator systems* (SMSs), also called *chasers* or *servicers* in the literature. An SMS consists of a satellite base equipped with one or more *robotic manipulators* (arms) with grappling devices on them and driven by a vision system which allows them to capture a *target (client)* satellite, or another object. An SMS also can be a large servicing manipulator mounted on a space facility.

Since the 1990s, the paradigm of on-orbit servicing using a SMS has attracted the interest of many researchers, see, for example, (Luo and Sakawa, 1990; Papadopoulos and Moosavian, 1994; Nagamatsu et al., 1996; Hirzinger, et al., 2000; Yoshida et al., 2006; Ma et al., 2007; Rekleitis, et al., 2007; Flores-Abad et al., 2017; Yoshida, 2003; Aghili and Parsa, 2007; Aghili and Parsa, 2009; Aghili, 2012; Aghili, 2020). These research works were motivated by several national and international missions not only for repairing, rescuing, and refueling failed satellites, but also for removal of defunct satellites or space debris (Kawamoto et al., 2003; Aghili and Turin, 2012b). Orbital debris removal using a SMS is also becoming of particular interest as space debris is on the rise, increasing the risk of collisions. Recently, the population growth has reached an unstable point in some congested orbits (Brachet, 2010). All these robotic servicing mission concepts require that a robotic arm captures the target in a safe and secure manner given operational and environmental constraints.

Targets for capture may be *cooperative*, i.e., a stable and safe target due to its operational Attitude and Orbit Control Subsystem (AOCS), or *non-cooperative* i.e., an unknown or tumbling target with a varying axis of rotation. They can also be *collaborative*, i.e., designed for capture or servicing, equipped with visual markers and grapple fixtures, or *non-collaborative,* as most of today’s satellites. In many cases in the literature, the term cooperative stands for collaborative, too.

As often revealed by ground observations, many on-orbit objects are tumbling in an uncontrolled way (non-cooperative targets), making the robotic capture a very challenging task (Kirchner G., et al., 2017). In fact, although several missions for on-orbit target capture using a SMS have been demonstrated so far, such as JAXA’s ETS-VII (Oda, 1999; Yoshida, 2003), DARPA’s Orbital Express (Whelan et al., 2000), and China’s Aolong-1 (Space Flight, 2016), robotic capture of a tumbling satellite has not been attempted yet.

The SMS for future on-orbit servicing missions will be operated from ground or autonomously, depending on mission constraints, requirements, and the level of technology readiness. Nevertheless, increased autonomy for robotic systems for on-orbit servicing missions is identified as the key technology by space agencies; it represents a critical challenge in space robotics (Center, 2010). This is especially true in the case of servicing non-cooperative targets, where teleoperation cannot be used due to time delays, communication dropouts, operator misperception, limited fields of view, and limited data bandwidth, as all work against a successful capture in a dynamic situation and make it an unsafe practice.

Clearly, only after a manipulator has successfully captured and stabilized a tumbling target, can a service operation be started. Therefore, a common robotic capture task for on-orbit servicing consists of four operational phases (Flores-abad et al., 2014a): a) observation and planning phase, b) final approach phase, c) impact and grasping/capture phase, and d) post-capture stabilization phase. From a different phasing perspective, the operation can be postulated (Aghili, 2013; Lampariello and Hirzinger, 2013) into three primitive operational phases: i) state and parameters estimation phase; ii) pre-grasping phase; and iii) post-grasping phase. Regardless of how to phase the operation, the primary challenges and key requirements for an end-to-end solution are the same.

To achieve safe and reliable OOS tasks, several challenges must be addressed, that render missions difficult and complex (Stieber and Fung, 1991; Flores-abad et al., 2014a). These include identifying a target prior to grasping, planning and control strategies to be employed, SMS performance during capture, and tackling the contact effects of an end-effector coming into physical contact with the target. To obtain a safe and well controlled contact operation, suitable hardware design approaches for manipulators and grippers, effective control methods, and well-planned operation procedures are all required.

Ground-based test and validation of perception and control systems for SMSs performing 3D contact operations is another key challenge in the presence of gravity as most of the large space manipulators cannot even lift themselves in the 1G environment. A number of experimental methods exist, such as suspension testing (Brown and Dolan, 1994), air -bearing supported testing (Schwartz et al., 2003), neutral buoyancy testing (Akin and Howard, 1992), and hardware-in-the-loop simulations (Ma et al., 2004; Ma et al., 2012). Addressing method limitations and increasing their scope is a prerequisite in boosting our confidence in their performance in space.

This paper provides a comprehensive review of research work in manipulation and capture on-orbit. *Dynamics of Space Robots in Orbit* discusses the dynamics of rigid and flexible elements of SMS in orbit, covering aspects such as the mode they operate in, the initial conditions, and modeling techniques. *Contact Dynamics* addresses the contact mechanics and motion behavior, both from the physical modeling and simulation viewpoints. *System Identification of In-Orbit Robotic Systems* is aiming at finding SMS and target properties, both of which are important for planning and control. Sensing of pose and state required for closed-loop control is discussed in *Sensing of Pose and State*. In *Motion Planning*, motion planning methods for grasping a target and on-orbit assembly are outlined. *Feedback Control* reviews feedback control methods and control issues for SMS during motion or interaction tasks. Ground testing testbeds as an essential prerequisite for capture operations are presented in *Ground Testbed Facilities*. Finally, several relevant missions and key technologies developed for capture of targets on orbit are presented in *Missions and Technology*.

## Dynamics of Space Robots in Orbit

**FIGURE 1:**
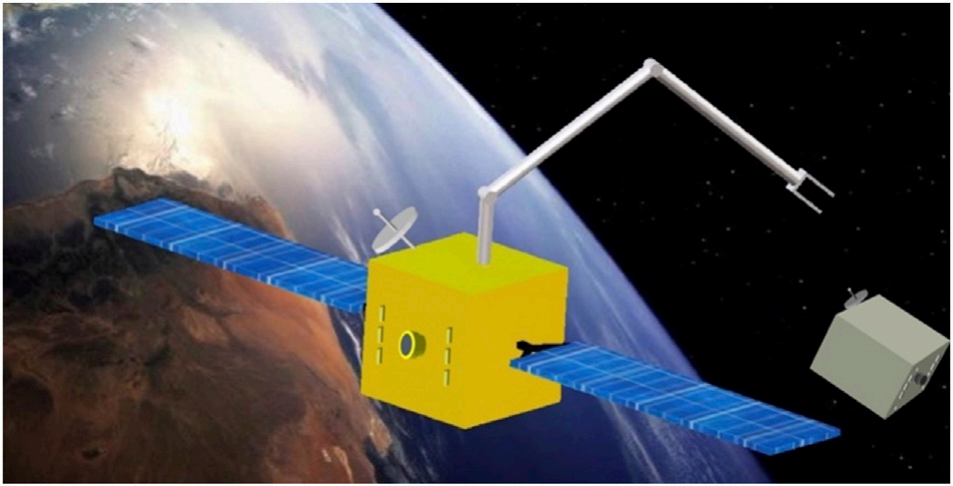
A SMS operating on-orbit (Nanos and Papadopoulos, 2017).

In-orbit space manipulator systems (SMS), see Figure 1, operate in a free-fall environment, where the gravitational effects are present during operations (Abiko and Yoshida, 2001). However, these effects, as well as non-gravitational existing perturbations such as thin air drag, magnetic force, and direct solar radiation pressure can be neglected due to the small-time scale of operations and the magnitude of the forces compared to operational forces (Dubowsky and Papadopoulos, 1993). Regarding spacecraft actuation, a SMS can operate in two main modes:

a) The *free-flying* mode, in which spacecraft thrusters are active. Then, the system Center of Mass (CoM) can translate. During this mode, magnetorquers or momentum control devices (MCD) such as reaction wheels or momentum gyros, can be active, too. This mode is employed during the final approach of a SMS to its target, so that the target is within the manipulator workspace.
b) The *free-floating* mode, in which external actions are excluded. As such, all spacecraft thrusters are turned off. Then, the system CoM cannot translate, while the spacecraft translates and rotates in response to manipulator motions. In some cases, the spacecraft attitude must be maintained during manipulator motions to avoid loss of communication with ground stations and solar panel disorientation. In this mode, the spacecraft attitude is controlled actively with momentum control devices (MCD), such as reaction wheels or momentum gyros, while the system CoM does not translate. If MCD are used then the mode is called *partial* free-floating. The free-floating or the partial free-floating modes are preferred during grasping, since they eliminate sudden motions due to thrusters, and conserve propellant and power.

SMS dynamics are important as they contribute to SMS analysis, to their effective simulation prior to deployment, and are needed in the development of advanced controllers. However, they tend to be computationally expensive; hence, methods for increasing the speed of computation are still needed.

### Free-Flying Space Manipulator Systems

To increase SMS mobility and perform larger end-effector displacements (Papadopoulos and Dubowsky, 1991b; Lampariello et al., 2003; Lampariello, R., 2013) or/and limit the contact force disturbances during docking operations (Shibli et al., 2005; Flores-abad et al., 2014a), the SMS must operate in the free-flying mode. In this mode, the spacecraft can be transferred and oriented arbitrarily in space. To achieve this, the initial designs were employing thrusters and MCDs controlled by the AOCS for spacecraft position and orientation control, and joint motors, controlled by a separate manipulator control system, for controlling manipulator functions. However, due to dynamic coupling, the motion of the manipulator affects the motion of the spacecraft and vice versa, creating undesired control disturbances (Seweryn and Banaszkiewicz, 2008; Misra and Bai, 2017; Christidi-Loumpasefski et al., 2020). Therefore, although initially each of these control systems would operate independently, the recent trend is to move towards a single and coordinated controller (see *Coordinated Control and Handling/Servicing Space Objects*).

The kinematics and dynamics of the system can be derived here using the spacecraft CoM as the representative point for the translational motion, following the so-called *direct path approach*, which in this case results in more compact equations of motion (Moosavian and Papadopoulos, 2004).

The end-effector linear velocity ${\dot{r}}_{\text{E}}$ and angular velocity $\omega_{E}$, both with respect to the inertial frame, are given by,

$$v_{E}={\left[\begin{array}{cc} {\dot{r}}_{E}^{T} & \omega_{E}^{T} \end{array}\right]}^{T}=J_{E}\left(\varepsilon ,n,\theta \right)\dot{q}$$ (1)

where $J_{E}$ is the system Jacobian matrix, depends on the spacecraft attitude expressed here with Euler parameters ε,n (to avoid possible existence of *representative singularities*) and on the manipulator configuration defined by the joint angles column vector θ and

$$\dot{q}={\left[\begin{array}{ccc} {\overset{\cdot }{r}}_{0}^{T} & \omega_{0}^{T} & {\overset{\cdot }{\theta }}^{T} \end{array}\right]}^{T}$$ (2)

where ${\overset{\cdot }{r}}_{0}$ and $\omega_{0}$ are the spacecraft linear and angular velocities, respectively. The joint angles column vector θ defines the manipulator configuration.

The system equations of motion for a free-flying SMS with *N* joints can be written as

$$H^{*}\left(\varepsilon ,n\mathrm{,\theta}\right)\overset{\cdot \cdot }{q}+c^{*}\left(\varepsilon ,n,\overset{\cdot }{\varepsilon },\dot{n}\mathrm{,\theta},\overset{\cdot }{\theta }\right)=Q$$ (3)

where the spacecraft attitude is defined by the Euler parameters ε,  n, $H^{*}$ is the system inertia matrix, $c^{*}$ is the nonlinear terms column vector, while the generalized forces vector is given by,

Q=Js[fsT0nsT0τT]T (4)

where fs0 and ns0 are the resultant forces and moments, respectively, acting on the spacecraft by the thrusters and the momentum devices, expressed in the spacecraft frame, τ is the *N*x1 column-vector of the joint torques and $J_{s}$ is an appropriate Jacobian matrix. Other methods to describe the spacecraft attitude, such as Euler parameters, can also be used (Paielli and Bach, 1993).

### Free-Floating Space Manipulator Systems

For small end-effector motions close to a target, the system may operate in the free-floating mode, during which an uncontrolled motion of the spacecraft arises because of the dynamic coupling between the spacecraft and the manipulator (Papadopoulos and Dubowsky, 1993; Wilde et al., 2018). Free-floating SMS exhibit nonholonomic behavior due to the non-integrability of the angular momentum conservation equation. Two cases are studied in the literature: a) zero initial angular momentum (Papadopoulos and Dubowsky, 1991a; Zong et al., 2020) and b) non-zero initial angular momentum (Nanos and Papadopoulos, 2010; Giordano et al., 2016).

#### Zero Initial Angular Momentum

In free-floating mode, no external forces act on the system, and therefore the system CoM is fixed in inertial space. Taking the *system* CoM as a representative point for the translational motion and using barycentric vectors (*barycentric vector approach*) which reflect both the geometric configuration and mass distribution of the system, decoupling of the total linear and angular motion from the rest of the equations, results (Papadopoulos and Dubowsky, 1991a). This allows the use of controllers for fixed-base manipulators, under some mild conditions (Papadopoulos, 1991).

In case of zero initial angular momentum, the angular momentum conservation yields (Papadopoulos and Dubowsky, 1991a),

D0(θ)ω00+Dq0(θ)θ⋅=0 (5)

where ω00 is the spacecraft angular velocity in the spacecraft frame and D0, Dq0 are inertia-type matrices.

The end-effector linear velocity ${\overset{\cdot }{r}}_{E}$ and angular velocity $\omega_{E}$, with respect to the inertial frame, are given by,

$$v_{E}={\left[\begin{array}{cc} {\overset{\cdot }{r}}_{E}^{T} & \omega_{E}^{T} \end{array}\right]}^{T}=J_{q}\left(\varepsilon ,n,\theta \right)\overset{\cdot }{\theta }$$ (6)

where the Euler parameters ε,n define the spacecraft orientation and $J_{q}$ is the generalized Jacobian matrix which depends on the dynamic properties (mass, inertia) of the free-floating SMS and reflects both momentum conservation laws and kinematic relations (Umetani and Yoshida, 1989). This matrix depends also on manipulator configuration θ and the spacecraft orientation which, as in the case of free-flying mode, it can be expressed by the Euler parameters ε,n. The generalized Jacobian matrix converges to the conventional Jacobian when the base body is relatively massive. The generalized Jacobian matrix converges to the conventional Jacobian when the base body is relatively massive.

Such systems are subject to path dependent Dynamic Singularities (DS) that complicate their path planning (Papadopoulos and Dubowsky, 1993), and restrict their effective workspace. To allow use of the entire SMS workspace, path planning methodologies allowing the end-effector to follow a desired path and avoiding DS have been proposed (Nanos and Papadopoulos, 2012) (also see *Singularity Avoidance*).

In the case of a free-floating SMS with *N* joints, *zero* angular momentum and negligible disturbances, the *reduced equations* of motion are (Papadopoulos and Dubowsky, 1991a):

$$\tau =H\left(\theta \right)\overset{\cdot \cdot }{\theta }+c\left(\theta ,\overset{\cdot }{\theta }\right)$$ (7)

where H(θ) is the *N* × *N* reduced inertia matrix and $c\left(\theta ,\overset{\cdot }{\theta }\right)$ is the *N* × 1 vector of non-linear terms of Coriolis and centrifugal forces.

Regarding the *partial free-floating mode,* in which the spacecraft attitude can be controlled with momentum devices only, the SMS equation of motion are (Papadopoulos, 1991)

[D0Dq0DqT0Dqq0][ω⋅00θ⋅⋅]+[c1c2]=[nrwτ] (8)

where $n_{\mathrm{rw}}$ is the net moment applied on the spacecraft by the momentum devices. Note that if $n_{\mathrm{rw}}=0$, Eq. 8 yields the *non-reduced* equations of motion for the free-floating SMS.

#### Non-Zero Initial Angular Momentum

Although *zero* initial system angular momentum is desired before using a SMS in free-floating mode, small collisions with the environment or on-off attitude controller inaccuracies result in small amounts of angular momentum. In principle, momentum can be absorbed using either thrusters or momentum control devices. However, thrusters by their nature use expendable propellants, limiting system life, and due to their on-off nature, they cannot reduce the angular momentum to zero. Momentum control devices require electrical power supplied by solar arrays; however, they tend to saturate and ultimately, also require use of thrusters for de-spinning. Therefore, in practice, a free-floating SMS can have small accumulated angular momentum.

A free-floating SMS with initial angular momentum is an affine system with a drift term (Yamada et al., 1995; Matsuno and Saito, 2001). This term is caused by the angular momentum and complicates the path planning and control of such systems (Nanos and Papadopoulos, 2015a; Nanos and Papadopoulos, 2017; Giordano et al., 2017; Giordano et al., 2018).

In case of non-zero initial angular momentum, the angular momentum conservation is given by (Nanos and Papadopoulos, 2010),

hCM=R0(ε,n)(D0(θ)ω00+Dq0(θ)θ⋅)=const. (9)

where $h_{\mathrm{CM}}$ is the constant non-zero angular momentum of the SMS expressed in the inertial frame and $R_{0}$ is the rotation matrix between the spacecraft frame and the inertial frame.

The end-effector linear velocity ${\overset{\cdot }{r}}_{E}$ and angular velocity $\omega_{E}$ are given by (Nanos, 2015):

$$v_{E}={\left[\begin{array}{cc} {\overset{\cdot }{r}}_{E}^{T} & \omega_{E}^{T} \end{array}\right]}^{T}=J_{q}\left(\varepsilon ,n,\theta \right)\overset{\cdot }{\theta }+J_{h}\left(\varepsilon ,n,\theta \right)h_{\mathrm{cm}}$$ (10)

where the Generalized Jacobian $J_{q}$ is not affected by the non-zero angular momentum. The end-effector linear/angular velocity is affected by the non-zero angular momentum via the additional term $J_{h}h_{\mathrm{cm}}$. Note that since the Generalized Jacobian $J_{q}$ is not affected by the non-zero angular momentum, the system DS configuration does not depend on the system initial angular momentum. This has allowed the development of path planning methodologies with which the end-effector can follow a desired path, avoiding DS in the presence of non-zero angular momentum (Nanos and Papadopoulos, 2015a; Rybus et al., 2016).

The reduced equations of motion of a free-floating SMS with non-zero angular momentum take the form (Nanos and Papadopoulos, 2010; Nanos and Papadopoulos, 2017):

$$\tau =H\left(\theta \right)\overset{\cdot \cdot }{\theta }+c\left(\mathrm{\theta ,}\overset{\cdot }{\theta }\right)+c_{h}\left(h_{\mathrm{cm}},\varepsilon ,n,\mathrm{\theta ,}\overset{\cdot }{\theta }\right)+g_{h}\left(h_{\mathrm{cm}},\varepsilon ,n,\mathrm{\theta ,}\overset{\cdot }{\theta }\right)$$ (11)

where the first two terms of Eq. 11 are the same as those in Eq. 7. The effect of the non-zero angular momentum on the system dynamics is included in term $c_{h}$, which is zero when the rates $\overset{\cdot }{\theta }$ are zero, and in term $g_{h}$ which does not vanish for zero joint rates $\overset{\cdot }{\theta }$, exhibiting characteristics similar to those of gravity terms in fixed base manipulators. Note that terms $c_{h}$ and $g_{h}$ are both functions of the spacecraft attitude described by the Euler parameters ε,n. Thus, the system’s reduced equations of motion depend on the spacecraft attitude.

Recently, the disturbances in the SMS response due to accumulated angular momentum of a rotating reaction wheel have been studied, with the aim of designing a controller compensating for such momentum disturbances (Christidi-Loumpasefski et al., 2020). In Mishra et al. (2020) the inertia-decoupled reduced Euler-Lagrange equations are exploited through the resulting block-diagonal inertia matrix to avoid the need for joint acceleration measurements in regulation tasks, in which a controller stabilizes the configuration of an orbital robot about a setpoint, in the specific setting that its spacecraft velocity is unmeasured. A well-partitioned Coriolis/centrifugal matrix is characterized by useful properties, which aid in the stability analysis.

### Flexible Space Manipulator Systems

In space applications, manipulator design differs from that in terrestrial applications. Due to the lack of gravity loading, SMS are designed to be lightweight and long reaching, which introduces link flexibilities. Moreover, lightweight, and flexible structures such as solar arrays, deployable truss antennas are employed (Du and Wang, 2020). Often, their joints are driven by harmonic drives for large gear ratio and compact design, introducing joint flexibility (Ulrich and Sasiadek, 2012). These types of flexibilities may cause vibrations both in the manipulator and the spacecraft during on-orbit servicing especially in tasks where physical contact occurs (Stieber and Fung, 1991; Schneider and Cannon Jr, 1992; Ma et al., 1997; Ma and Wang, 2007).

Considering the gearmotor dynamics and using the *barycentric vector approach*, the angular momentum conservation for a free-floating SMS, is given by (Nanos and Papadopoulos, 2015b):

hCM=R0(ε,n)(D∗0ω00+Dq*0θ⋅+Dθm0θ⋅m) (12)

where the column vector $\theta_{m}$ defines motor side angular positions. Motor variables are introduced to allow for joint flexibilities, i.e., different motor-side and link-side angles. The terms D∗0,Dq*0,Dθm0 are inertia-type matrices. The contribution of the motor dynamics on the SMS angular momentum is given by the term Dθm0θ⋅m.

Assuming that all system flexibilities are lumped to *joint flexibilities*, it can be shown that for a free-floating SMS, the link and motor equations are not only dynamically coupled through the joint elastic torques, but also at the acceleration level (Nanos and Papadopoulos, 2015b):

$$\begin{array}{l} H_{\mathrm{qq}}\left(\theta \right)\overset{\cdot \cdot }{\theta }+H_{q\theta_{m}}\left(\theta \right){\overset{\cdot \cdot }{\theta }}_{m}+c_{1}\left(\theta ,\overset{\cdot }{\theta },{\overset{\cdot }{\theta }}_{m}\right)-K\left(\theta_{m}-\theta \right)-B\left({\overset{\cdot }{\theta }}_{m}-\overset{\cdot }{\theta }\right)=0\\ H_{q\theta_{m}}^{T}\left(\theta \right)\overset{\cdot \cdot }{\theta }+H_{\theta_{m}\theta_{m}}\left(\theta \right){\overset{\cdot \cdot }{\theta }}_{m}+c_{2}\left(\theta ,\overset{\cdot }{\theta },{\overset{\cdot }{\theta }}_{m}\right)+K\left(\theta_{m}-\theta \right)+B\left({\overset{\cdot }{\theta }}_{m}-\overset{\cdot }{\theta }\right)=\tau \end{array}$$ (13)

where $H_{\theta_{m}\theta_{m}},H_{\mathrm{qq}},H_{q\theta_{m}}$ are inertia—type matrices while the vectors $c_{1}$ and $c_{2}$ contain the nonlinear terms of centrifugal and Coriolis forces. The matrices **K** and **B** are the stiffness and damping matrices, respectively, which describe the joint flexibilities. The coupling between motor and link accelerations is given via the matrix $H_{q\theta_{m}}$. It has been shown that the structure of this matrix allows the design of trajectory tracking controllers, both in joint and Cartesian spaces, with small computational effort (Nanos and Papadopoulos, 2015b).

A different modelling approach called the *singular perturbation* method has been proposed for the case the joint stiffness is relatively large, but still finite (Hu and Vucovich, 1997). Then, the system exhibits a two-time scale dynamic behavior in terms of rigid and elastic variables. Using this method, one can apply controllers which consist of a slow control action designed based on a rigid robot model, and a fast control action designed to damp the joint elastic oscillations (Yu and Chen 2014).

The assumption that all system flexibilities are lumped to joint flexibilities is reasonable for SMS with short links. However, in some cases the design of lightweight and long reach manipulators is strongly preferred as it reduces launch mass and increases manipulator reach. A problem of such lightweight space manipulators is the increased structural flexibility of the links, which causes structural vibrations. Flexible links can be modeled as Euler-Bernoulli beams (Green and Sasiadek, 2007; Christidi-Loumpasefski et al., 2020) and a finite-dimensional model of link flexibility can be obtained by the assumed modes technique (Zhang et al., 2020).

## Contact Dynamics

**FIGURE 2:**
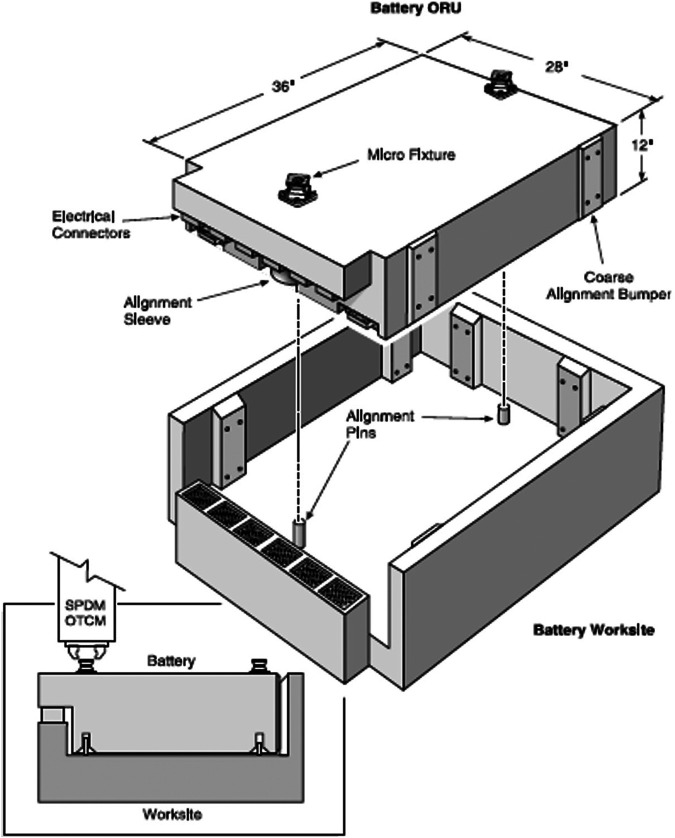
An ISS battery and housing with three peg-in-hole contact pairs (Ma and Carr, 1998).

All capture and some manipulation operations involve physical contact between the robot and an external object or the environment. Contact operations are among the most difficult operations for a robot, whose contact behavior is governed by contact dynamics. Although contact dynamics of individual rigid or elastic bodies has been extensively studied in the last few decades (Gilardi and Sharf, 2002; Flores and Lankarani, 2016; Natsiavas, 2019), accurate modeling and control of contact behaviors of multibody systems are still challenging for most robotic operations in space, especially when contact interfaces are complex as commonly seen in on-orbit servicing (Flores-abad et al., 2014a), orbital debris removal (Shan et al., 2016) and on-orbit assembly (Piskorz and Jones, 2018). For example, Figure 2 shows a typical battery and its housing structure on the International Space Station (ISS) whose contact geometry is characterized as three cascade peg-in-hole pairs (one rectangular peg/hole and two cylindrical pegs/holes). This is just one of many batteries of different sizes and designs on ISS, which have been maintained by either EVAs or the dual-arm ISS robot Special Purpose Dexterous Manipulator (SPDM). It was found that the insertion or removal of such a battery into or from its housing worksite was one of the most difficult operations of the ISS robot and hence extensive research had to be done to ensure success of these operations (Stieber and Fung, 1991; Ma and Carr, 1998). Therefore, modeling, simulation and verification of contact dynamics and control approaches are always among the most critical parts of space robot development and operations.

In practice, contact often occurs among mechanical parts with complex geometries of convex and concave mixed topology, which cannot be simply represented by two regular shapes or point-plane contact. The most used modeling method is the surface compliance-based approach (Ma, 1995; Gilardi and Sharf, 2002). With this method, first step is to fully understand how the two contact bodies will contact and engage in the robotic operation. With such an understanding, one can then partition each contact body into many small enough sub-bodies or surfaces, so that all the contacting areas or points, especially these in the concave surfaces, can be accurately represented (Ma, 2000). The next step is to identify all the possible contacts and calculate the geometrically overlapped contact regions between sub-bodies based on the simulated motion states of these contact bodies in the robotic multibody system.

Many CAD or computer graphics algorithms are available for efficient calculation of contact regions (Choi et al., 2010). The final step is to calculate contact forces for all the contact regions. At each contact region, there is a normal force along the surface normal, a material/structural damping force also in the normal direction, and a friction force in the tangential plane of the contacting surfaces (Ma, 1995; Gilardi and Sharf, 2002; Gonthier et al., 2004). Most of the normal contact force models are based on linear spring–dashpot or nonlinear Hertzian spring–dashpot laws with damping terms to accommodate the energy loss (Machado et al., 2012). The traditional one-dimensional Coulomb friction model does not work well for simulating general 3D sticking (jamming) or stick-slip phenomena. This problem was addressed by introducing a 3D bristle friction model in Liang et al. (2012).

Contact dynamics simulations for practical cases with stiff contact materials and complex contact geometries (e.g., manipulator capturing or spacecraft docking) usually are very inefficient because of the required large number of iterative computations and very small numerical integration step size (for numerical stability). Many studies have been devoted to improving efficiency of computational contact models. Mazhar et al. (2015) presented a solution method to simulate the multibody systems with frictional contact. The presented method reduced the required time by one or two orders of magnitude. Navarro and de Souza Braun (2013) determined the normal spring stiffness coefficient of a linear normal contact model through numerical solutions for the overlap between particles in non-linear models.

Boos and McPhee (2013) proposed a volumetric contact dynamics model for the purpose of generating reliable and rapid simulations of contact dynamics, which allows modeling of contact between complex geometries and relatively large contact surfaces, while being less expensive computationally than finite element methods. Ma and Wang (2007) and Liang et al. (2011) presented a method to combine linearized contact force terms with the manipulator’s structural stiffness and damping matrices for model order reduction. The method can improve simulation speed by one or more orders but applies to flexible manipulators with slow motion cases only. Askari (2021) introduced a concept to simulate either soft or conformal contacts and developed mathematically closed-form contact models, which are easy-to-implement while resolving the discontinuity issue with the Kelvin-Voigt model.

A unifying dynamics formulation for nonsmooth multi-body systems subject to changing topology and multiple contacts based on a linear projection operator was presented in Aghili (2019). It follows by development of an energetically consistent model of slipping and sticking frictional impacts for robotic systems in contact with a frictional surface in Aghili (2020). This work reveals that a contact dynamics model can lead to energetic consistency in both slip and stick states upon imposing specific constraints on the coefficient of friction and the coefficient of restitution.

Zhao et al. (2016) developed a multi-point rigid-body contact dynamics model which calculated contact forces using kinematic constraints and Lagrange multipliers. They found that, when the model was applied to an APDS (androgynous peripheral docking system) docking case, the resulting simulation was more efficient than surface-compliance based models. Ma et al. (2020) developed an inverse research strategy towards the establishment of contact force model for complex contacting surfaces by utilizing parameter identification methods. Wang and Liu (2020) revealed the shortcoming of the improved contact stiffness coefficient and developed two different contact force models for the internal and external contact forms associated with the hysteresis damping factor from the Lankarani-Nikravesh contact force model (L-N model). Even with these recent developments regarding model efficiency, real-time contact dynamics simulation for realistic contact operations is still difficult to guarantee due to complex contact geometry and variable numerical integration step size for solving stiff differential equations.

All contact dynamics models for practical capture missions will have many model parameters describing the geometry, stiffness, friction, and material damping properties of the contact interfaces. Accurate identification of these model parameters remains a challenge. Although research efforts have been made for identifying model parameters from hardware tests (Weber et al., 2006; Kim and Ma, 2007; Verscheure et al., 2008), most of the users still have to assume or estimate parameters based on design data. Even if one can identify experimentally these parameters from real contact parts before launch, the parameter values can still change significantly in space due to changing of material properties and the operational environment in space. To address these uncertainties, Liu et al. (2020) proposed a hybrid contact modeling methodology to combine a traditional analytical contact model with a data-driven neuron network model, where the analytical model represents theory-based general contact dynamics, while the neuron-network based data-driven model captures the existing known and unknown unmodelled errors and uncertainties. The data driven model can be trained using machine learning techniques from experimental data collected from repeated testing of real contact hardware, which must be done anyway for all the space systems before they are launched to the space. More research is needed to mature this new hybrid modeling approach. Another area of contact modeling requiring significant further research is the frictional contact problem with large deformation because of the increasing development and applications of soft grasping (e.g., robotic handling of soft/fabric covers of a satellite) or soft robots (Botta et al., 2017; Sun et al., 2021; Sadati et al., 2021).

Again, due to high uncertainties in contact dynamics modeling and simulations, space agencies across the world developed various hardware-in-the-loop (HIL) simulation facilities to test and verify critical contact operations of space robotic systems before they are launched into space. NASA/MSFC first developed an HIL simulation facility to test Space Shuttle docking or berthing to ISS (Tobbe et al., 1991). Canadian Space Agency (CSA) developed SPDM Task Verification Facility (STVF) to test ISS robot SPDM critical contact operations (Piedboeuf et al., 1999; Ma et al., 2004; Aghili, 2019). German Aerospace Center (DLR) developed the European Proximity Operations Simulator (EPOS) to test satellite rendezvous and docking operations for the DEOS and OLEV missions (Boge and Ma, 2011). China Academy of Space Technology (CAST) developed a Manipulator Task Verification Facility to test Chinese space station manipulators on-orbit service operations (Mou et al., 2018). US Naval Research Laboratory (NRL) developed a test facility to test robotic operations for the RSGS mission (Roesler et al., 2017). NASA Goddard Space Flight Center (GSFC) also developed a test facility to test the Space Infrastructure Dexterous Robot (SPIDER) on-orbit servicing operations for the OSAM-1 mission (NASA/GSFC, n.d.). Details of these major facilities are discussed in Section 8.3.

## System Identification of In-Orbit Robotic Systems

As system properties may change during operations in space, methods are needed to establish these properties on-orbit for health monitoring, planning and control purposes. Research efforts have focused on the development of methods for rigid satellites, while other efforts have concentrated on the identification of flexible satellites, i.e., satellites with flexible appendages. Both are important elements of satellite (or debris) capture operations, as they reduce the operational risks and allow tuning of the control parameters involved.

### Rigid Satellites and Tumbling Objects

To reduce the risk of a defunct rigid satellite capture by a SMS, researchers have proposed methods to identify its parameters in the *pre-capture phase* while others have developed methods that require the capture phase to be accomplished first, i.e., for the *post-capture phase*. The methods developed for the identification of rigid satellites in the pre-capture phase can be classified as *vision-based* and *momentum based*. Vision-based methods are addressed in detail in *Vision-Based State/Inertia Parameter Estimation and Motion Prediction*. However, methods relying on vision alone cannot identify all the individual inertia parameters. They estimate the ratios of the moments of inertia, the CoM location, and the orientation of principal axes, only.

To identify the full inertia matrix, momentum-based methods were developed, in which the servicing satellite applies forces and moments to the target. Sheinfeld and Rock (2009) first proposed the preliminary concept; Christidi-Loumpasefski and Papadopoulos (2018) extended it to address the full identification issue, followed by experimental verification. Meng et al. (2019) also proposed the application of an impulse to the satellite by making soft contact using a flexible fish-rod like sticker mounted on the SMS, and the use of data from visual and force sensors to estimate the tumbling motion (pose and linear/angular velocities) and identify all ten inertial parameters (mass, CoM location, and moments and products of inertia) of the satellite. Their simulation study showed that for an object weighing 1,000 kg, only a small force of less than 10 N is sufficient to accomplish the task of identifying all inertial parameters. To avoid physical contact, Meng et al. (2020) showed the feasibility of applying non-contact impulse using eddy current to identify all ten inertial parameters. In the same work they demonstrated experimentally that the method can be used for pre-capture detumbling of a tumbling target, so that the high risk of physical contact with the tumbling object for the capture phase is avoided.

Methods developed for the post-capture phase can be classified into those that use the *equations of motion* and those based *on momentum equations*. Murotsu et al. (1994) estimated the inertial parameters of an object captured by a space robot based on the equations of motion derived by the Newton-Euler approach, under the condition that the robot is free-floating. Lampariello and Hirzinger (2005), proposed a method for the identification of the base body and load on the end-effector, using accelerometers. Rackl et al. (2013) addressed problems in the SMS satellite identification, the captured satellite or both, using direct robot joint torque sensing. The methods based on the equations of motion, require acceleration measurements, which are very noisy. However, if torque sensors at the robot joints are available, the use of acceleration measurements can be avoided, to the advantage of the accuracy of the identification process (Rackl et al., 2013). This approach was shown to be more accurate than momentum-based identification methods.

To avoid noise corrupting estimates, several researchers formulated momentum-based identification methods. Murotsu et al. (1994) focused on estimating the inertial parameters of an object captured by a space robot based on the conservation of momentum, under the condition that the robot is free-floating. Ma et al. (2008) made use of a robotic arm to change the inertia distribution of a spacecraft system. Considering measurable velocity changes and computable inertia changes of the robotic arm, the inertia parameters of the spacecraft body were identified. Nguyen-Huynh and Sharf (2013) developed an online momentum-based estimation method for inertial parameter identification of an unknown tumbling target. Chu et al. (2017) estimated the inertial parameters of a captured satellite using contact force information. Xu et al. (2017), proposed a method that uses both equations of motion and momentum equations for identifying all inertial properties of a captured satellite (Murotsu et al., 1994).

### Flexible Satellites

Satellites are often equipped with flexible appendages and their identification is well established. Rackl and Lampariello (2014) addressed the effects of flexible appendages to the free-floating dynamics and to the rigid body parameter identification of a SMS satellite/base. A lumped parameter model was used for the flexible appendages and a method to identify its parameters was presented. However, flexible satellites are more often considered as distributed models and are identified based on modal analysis algorithms. Modal analysis of flexible components is studied particularly well for structural applications such as those in civil engineering and extensive literature in this field is available; examples of spacecraft applications exist as well. On-orbit identification experiments of structural modal parameters have been implemented on some spacecraft such as the Hubble Space Telescope (HST) (Anthony and Andersen, 1995), the Galileo spacecraft (Pappa and Juang, 1984), and the Engineering Test Satellite VIII (ETS-VIII) (Kasai et al., 2009). Accelerometer data from the ROSA flight experiment on the ISS were analyzed to identify the ROSA system modal parameters (Chamberlain et al., 2018).

### System Identification of Space Manipulator Systems

Space manipulator parameters and kinematics/dynamics models are reasonably understood and measured before launch to space. However, some of the parameters may change in orbit and hence, on-orbit identification or calibration of model parameters is needed. Several researchers have developed methods for rigid SMS, while others have studied the identification of flexible SMS, i.e., SMS with flexible joints and/or flexible links.

#### Rigid Space Manipulator Systems

The methods developed for the identification of rigid SMS are mainly momentum-based. Yoshida and Abiko (2002) used estimation errors for the reaction wheel momentum to compute the deviations of the parameters of a SMS from the nominal ones. Xu et al. (2017) proposed a method that uses both equations of motion and momentum equations for identifying all inertial properties of each body of a SMS. Christidi-Loumpasefski et al., (2017) proposed a method that allows identification of all system parameters required to reconstruct the free-floating joint-space dynamics of a SMS, based on the angular momentum conservation. This method was enhanced further to identify all system parameters required to reconstruct the free-floating Cartesian-space dynamics of a SMS, based on the angular momentum conservation and kinematics equations (Christidi-Loumpasefski et al., 2020). Naveen et al. (2019) developed a momentum-based method that identifies all parameters required to reconstruct the free-flying dynamics of a SMS, using the linear and angular momentum equations.

#### Flexible Space Manipulator Systems

Α challenge in the design of space robotic manipulators is to use light materials, suitable for typical on-orbit tasks. Lightweight structures improve the payload-to-arm mass ratio. A drawback of such lightweight manipulators is the increased link structural flexibility. SMS are subject also to joint flexibilities that arise when motion transmission elements such as harmonic drives, transmission belts and long shafts are used. Both types of flexibilities cause vibrations, which are profound when manipulating large payloads; if neglected, poor performance and even control instabilities may result. To tackle flexibilities issues, advanced control strategies are required; however, these need knowledge of system parameters.

Krzyżak et al. (2012) studied the modeling and identification of two-degree-of-freedom (DoF) planar SMS with flexible joints by block-oriented systems. The joint dynamics included non-linear stiffness and friction terms. The manipulator was represented by a Hammerstein model consisting of a memoryless nonlinearity followed by a dynamic linear system. Zhiyu et al. (2019) linearized the dynamic model of a two-DoF planar SMS with flexible links at an arbitrary working point and studied the estimation of the system state-space model during the capture of an unknown object. A recursive tracking approach based on the recursive predictor-based subspace identification algorithm was proposed to identify the manipulator payload mass parameter. Nanos and Papadopoulos (2019) studied the estimation of the full dynamics of a spatial SMS with flexible joints. It was shown that methods based on the angular momentum conservation, which are tolerant to sensor noise, cannot estimate joint flexibility parameters.

A new parameter estimation method, based on the energy balance during the motion of a flexible joint SMS, was developed. The method estimates all system parameters including those that describe the joint flexibilities, requiring measurements of joint angles and rates, spacecraft attitude and angular velocity, and joint torques. Christidi-Loumpasefski et al. (2020) further enhanced the study, applying the energy balance method to the estimation of link flexibility parameters in addition to all SMS inertial and joint flexibility parameters.

Although many identification methods have been developed, difficulties for identifying model parameters of a SMS in operation including flexible and rigid elements, hard nonlinearities, and perhaps sloshing effects, still require further research efforts.

## Sensing of Pose and State

### Motion State Estimation

Robust relative navigation systems are critical for many current and near-future lunar or space exploration missions to support rendezvous, proximity operations and docking for both crewed and uncrewed vehicles. Reliable relative pose information in full 6-DoF is required during approach and docking of a visiting vehicle with the ISS. It is deemed that the safety of the controlled spacecraft during such proximity maneuvers critically depends on the performance and robustness of the relative navigation systems. Their failure to provide continuous and accurate pose (position and orientation) is considered as a critical hazard or even a catastrophic hazard that can cause failure of the mission all together. This is a challenging issue that must be addressed properly prior to the routine deployment of SMS in orbit.

Several relative navigation sensors exist capable of providing measurements for estimating the pose of objects having relative motion. Application of radar and altimetry for space-borne navigation systems begun more than half a century ago (Kriegsman, 1966), while X-ray pulsars for relative navigation between two spacecraft in deep space was introduced (Emadzadeh and Speyer, 2011; Liu et al. 2015). Other relative navigation methods focus on using Global Position System (GPS) for determining both absolute and relative position between two spacecraft (Wolfe and Speyer, 2004).

Vision systems have been developed capable of estimating the pose of two objects moving with respect to each other. Among them, an active vision system such as a Laser Camera System (LCS) is preferable because of its robustness in the harsh lighting conditions of space (Samson, et al., 2004). Although using radar or GPS for relative navigation systems are with the advantage of long-range distance measurement, they have less resolution and precision compared with vision-based systems. Moreover, the advent of relatively low-cost and commercially available laser range sensors and scanners, which has been greatly exploited for autonomous navigation of robotic vehicles (Lu and Tomizuka, 2006) makes them preferred sensor of choice in relative navigation systems. A rendezvous laser radar was used as the primary navigation to perform unmanned autonomous rendezvous docking experiments in the ETS-VII mission (Mokuno et al., 2004). Vision algorithms for laser scanners have been also developed for motion estimation of free-floating objects to support a variety of on-orbit proximity operations (Masutani et al., 1994; Hillenbrand and Lampariello, 2005; Aghili and Parsa, 2008). In Lingenauber et al. (2017) the potential benefits of plenoptic cameras for robot vision during on-orbit servicing missions were discussed.

The conventional vision-based pose estimation algorithms are essentially 3D registration processes, by which the range data collected from different views are aligned in a common coordinate system. The *iterative closest point* (ICP) is the cornerstone of 3D vision-based pose estimation algorithm. The iterative procedure minimizes distance between a point cloud in one dataset and the closest points in the other (Besl and McKay, 1992; Greenspan and Yurick, 2003). Typically, one dataset is a set of 3D point-cloud acquired by scanning an object, while the other one is a model set such as a CAD model of the same object. The basic ICP algorithm has proven to be very useful in the processing of range data (Greenspan and Yurick, 2003). Subsequently, several variations on the basic method have been developed to optimize different phases of the algorithm (Greenspan and Yurick, 2003). Convergence of ICP iterations and the accuracy of the fine alignment process depends on quality of the 3-D vision data that can be adversely affected by many factors such as sensor noise, disturbance, outliers, symmetric view of the target, or incomplete scan data.

A review of collaborative and non-collaborative spacecraft pose determination techniques for close-proximity operations can be found in Opromolla et al. (2017). Approaches in visual tracking of a non-collaborative as well as a partially collaborative satellite, to enable close-range rendezvous between a servicer and a target satellite, were presented in Oumer (2016). Experimental results indicate that camera-based methods provide robust and accurate tracking for the approach to malfunctioning satellites in spite of the difficulties associated with specularities and direct sunlight (Oumer, 2016; Lampariello et al., 2021). Taking advantage of the simple dynamics of a free-floating object, which is not acted upon by any external force or moment, researchers have employed different observers to track and predict the motion of free-floating space objects (Hillenbrand and Lampariello, 2005; Aghili and Parsa, 2007). However, relative thrust acceleration was not accounted for, and therefore these methods are not applicable for relative navigation. A robust 6-DoF relative navigation by combining the iterative closet point (ICP) registration algorithm and a noise - *adaptive Kalman filter* (AKF) in a closed-loop configuration together with using measurements from a laser scanner and an inertial measurement unit (IMU) was presented in Aghili and Su (2016).

### Vision-Based State/Inertia Parameter Estimation and Motion Prediction

Visually guided robotic capture of a moving object often requires long-term prediction of the object motion not only for a smooth capture but also because visual feedback may not be continually available, e.g., due to vision obstruction by the robotic arm. The use of laser range data has been proposed for motion estimation of free-floating space objects (Lichter and Dubowsky, 2004; Hillenbrand and Lampariello, 2005; Aghili and Parsa, 2007; Aghili and Parsa, 2009). Lichter and Dubowsky (2004) employed two separate Kalman filters for the rotational and translational dynamics of a free-floating space object to reduce the noise of a range sensor. Since principal inertia of the target are directly included in the state vector to be estimated by a *Kalman Filter* (KF), a normalization and re-parameterization of the estimated inertia values must be performed at every step of the KF cycle. Hillenbrand and Lampariello (2005) developed a nonlinear least-squares estimation method for long-term motion prediction (<100 s) and for the model identification of a free-tumbling space object. The identification process estimates the six inertia parameters of the target inertia tensor (with respect to an arbitrary constant factor) and the target’s velocity at the initial time of the motion prediction, which is then solved as an Initial Value Problem in the camera frame. Aghili and Parsa (2007, 2009) developed a computationally efficient, noise AKF for the motion estimation and prediction of a free-tumbling target satellite. The filter receives noisy pose measurements from a laser vision system aboard the chaser satellite at a close distance in a neighboring orbit, and estimates the full states, all the inertia parameters of the target satellite, as well as the covariance of the measurement noise. This motion estimation/prediction scheme was further developed for a fault-tolerant pose estimation of space objects (Aghili et al., 2011; Aghili and Su, 2016). The robustness and accuracy of fault-tolerant pose estimation was demonstrated through a hardware-in-the loop simulation setting.

Identification of states and parameters of space objects using minimum set inertial parameters, i.e, in terms of two relative inertia variables, was presented in Aghili (2013). Tweddle et al. (2015), developed a vision-based method that can estimate some of the satellite’s inertial properties. In Setterfield et al. (2018), a procedure for estimating the inertial properties of a passive in-orbit object was presented, in which the principal axes and inertia ratios of the object were estimated using an explicit comparison between the estimated and an analytically predicted body-frame angular velocity. The angular velocity was estimated with finite differences. The method was applied on the *Synchronized Position Hold Engage and Reorient Experimental Satellites* (SPHERES) and the *Visual Estimation for Relative Tracking and Inspection of Generic Objects* (VERTIGO) test platform on the ISS with two fast multi-axis tumble trajectories. In Lampariello et al. (2021), the nonlinear least-squares method in Hillenbrand and Lampariello (2005) was extended to perform long-term rotational motion prediction, up to <600 s. The method was compared to a constrained least-squares approach (Benninghoff and Boge, 2015) and to the *Extended Kalman Filter* (EKF) method in Aghili and Parsa (2009), showing a better performance in critical tumbling states of the target satellite, such as the condition close to a flat spin. Meng et al. (2019) investigated the use of EKF to estimate motion state from noisy vision images as a part of the vision and impulse combined solution for identifying all the inertial parameters. They proposed a scheme of decaying process noise for the covariance matrix, leading to both fast and accurate convergence of the estimate.

The state estimation of the chaser satellite also was recognized in Gallardo et al. (2019) to be of great relevance for the control of a free-floating impedance controller during the capturing phase. In fact, the interfacing of a fast-sampled robot controller with a slow sampled *Guidance, Navigation and Control* (GNC)-bus on the spacecraft causes performance loss for the robot controller. Additionally, only slow-sampled, and noisy exteroceptive sensors which provide relative pose measurements, may be available for feedback. As such, an EKF was presented which, based on IMU and star/sun trackers sensors, as well as on the slow-sampled poses of the tumbling target derived from visual camera and LIDAR sensors, computes a fast, full state estimation of both servicer and target. A similar task was addressed in Mishra et al. (2019) with a nonlinear observer, which estimates the inertial pose and the velocity of a free-floating non-cooperative target using only relative pose measurements. A novel dynamics model in terms of *minimum set* inertia parameters was developed in Aghili (2021) that was utilized to design a constrained and adaptive EKF for estimation of not only the states and parameter but also the covariance of the vision sensor noise. This work has demonstrated that incorporation of the minimum set of inertia parameters in the estimator internal model elevates the system degree of observability.

## Motion Planning

Spacecraft guidance provides reference trajectories and attitude profiles for the final approach phase of a SMS to reach a berthing point and start capture, docking and servicing operations. Similarly, manipulator motion planning provides joint and end-effector trajectories necessary to grasp a target, mate appropriate interfaces, or perform *orbital replacement unit* (ORU) exchanges (Dubanchet, et al., 2020). Due to parameter errors and uncertainties, a feedback control, as discussed in *Feedback Control*, is also needed to complete a capture operation. In the following subsections, the capture of a tumbling target satellite is addressed first, followed by the on-orbit assembly of a large space telescope.

### Capture of a Tumbling Target

**FIGURE 3:**
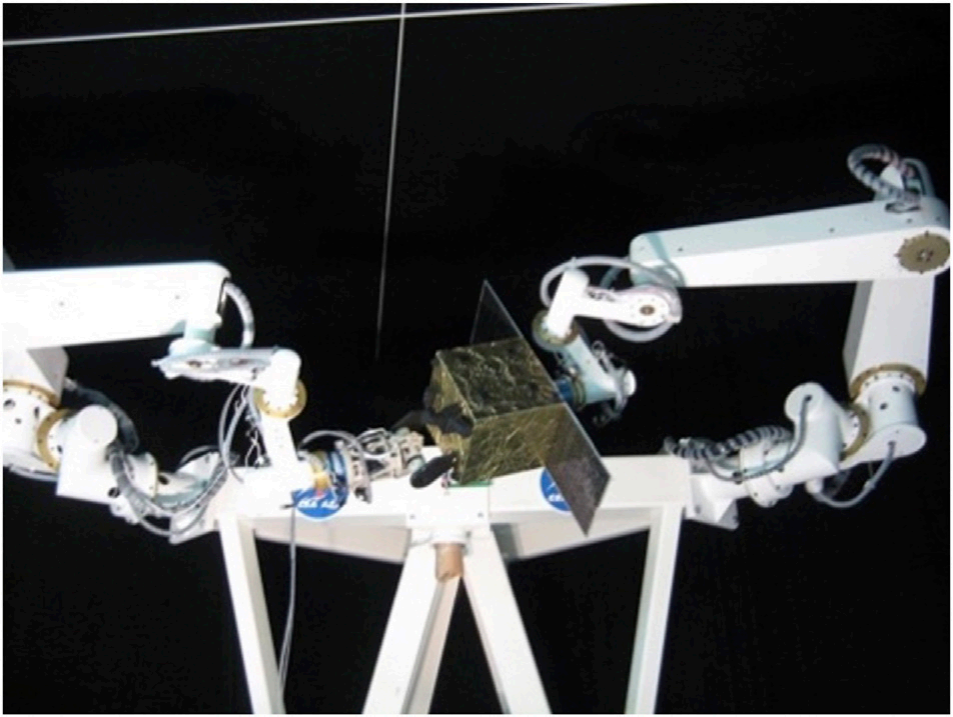
Capture of a tumbling satellite on the CSA dual-arm simulator testbed (CSA).

The task of capturing a free-tumbling target satellite is typically divided into the following steps, as already described in *Introduction* (see also Figure 3): a state and parameters estimation phase; a pre-grasping phase; a grasping phase, and a post-grasping phase. The pre-grasping phase can be further divided into an approach maneuver of the chaser to a predefined Mating Point near the target; and a pre-grasping maneuver of the robotic arm on the chaser satellite, to move the robot end-effector onto the capture point of the target.

The grasping itself includes soft grasping, during which the chaser and target cannot move away of each other, and hard grasping during which rigidization occurs, see also *Arms, Grippers, and End-Effectors*. As such, the post-grasping phase first involves a maneuver of the robotic arm on the chaser satellite to stabilize the chaser-target stack (rigidization). The detumbling of the chaser-target stack then follows. The latter is addressed in *Post-Grasping Motion Planning* and in *Capturing/Contacting a Target—Impedance/Compliance Control* and *Coordinated Control and Handling/Servicing Space Objects* as a control problem. The pre-grasping phase requires as input the prediction of the tumbling target’s motion (the prediction task is addressed in *Vision-Based State/Inertia Parameter Estimation and Motion Prediction*), as postulated in Jacobsen et al. (2002), Aghili (2013) and Lampariello and Hirzinger (2013). The guidance of an SMS to rendezvous and capture a tumbling free-floating object in a safe and secure manner remains a challenging task today.

#### Chaser Approach Maneuver

The approach maneuver of the chaser to a predefined Mating Point belongs to the AOCS domain, see *Free-Flying Space Manipulator Systems*. However, free-flying and AOCS tasks tend to overlap, as also described in *Coordinated Control and Handling/Servicing Space Objects* for feedback control. As such, some pointers are provided here, which may serve as an introduction to the subject.

With a target locally stationary, the approach phase can be achieved by point-to-point planning and depending on the actuation mode, by simple on-off thruster control, and attitude fine-tuning using momentum exchange devices. Capturing a tumbling, non-cooperative target is more challenging, as here velocity matching between the SMS end-effector and the capture point is required. The chaser approach maneuver can be performed with or without synchronization of the chaser motion with that of the target. An example of the former approach can be found in ESA’s e.Deorbit scenario, while of the latter in DLR’s DEOS scenario (see *Missions and Mission Studies*).

In Jacobsen et al. (2002) a simplex numerical optimization approach is applied to the chaser approach problem, with particular emphasis on safety metrics, such as time to collision. An optimal trajectory was proposed in Ma et al. (2007), in which an iterative algorithm, stemming from an indirect formulation of the optimal control problem for a planar case, was proposed for minimum time and fuel consumption. An extension of this work (Boyarko et al., 2011) considers the full 6-DoF proximity motion dynamics. A guidance method for fuel-optimal trajectories (Breger and How, 2008) employed mixed-integer linear programming (MILP). A simple and widely used algorithm for real-time trajectory planning is the glidescope algorithm (Hablani et al., 2002), which is based on the closed-form solution of the linear Clohessy–Wiltshire equations. A hybrid linear quadratic regulator/artificial potential function (LQR/APF) scheme for the guidance and control of multiple spacecraft proximity maneuvers, was proposed in Bevilacquaa et al. (2011), while methods for guidance and control of a SMS approaching a non-cooperative target were developed in the presence of uncertainty and measurements incompleteness (Somov et al., 2018).

The use of convex programming techniques is another common choice in aerospace guidance and control applications, as described in the survey (Liu, 2014). A convex programming-based guidance scheme (Misra and Bai, 2017) and an optimization technique for the pre-capture trajectory (Aghili, 2009) were proposed, where only the SMS base attitude is controlled (partial free-floating mode), removing the nonholonomic characteristics of the system. In MacPherson et al. (2018), an optimal control strategy to exploit the dynamic robustness of gecko-inspired dry adhesive grippers for the task of grasping a free-floating, spinning object is presented. The spacecraft rendezvous guidance problem was also tackled in the convex programming-based context in Virgili-Llop et al. (2017, 2019), where convexification was applied to the collision avoidance constraints, deriving from the solar appendages of the target. The resulting motion planning method is proven to converge always to a stationary point, independently of the initial guess, in short computation time. The same authors however recognized that the addressed optimization problem still exhibits local minima and propose finding a good minimum with multiple calls of the motion planner online. In Stoneman and Lampariello (2016) emphasis was given to collision avoidance, which was shown to play an important role in the e.Deorbit scenario (see *Missions and Mission Studies*). The trajectory planning problem is formulated as a state-constrained, nonlinear program (NLP) and solved for many planning queries offline, to provide a set of (close to) globally optimal solutions. This set of solutions can then be used to warm-start the motion planner in an online setting, via regression. A description of a mission pipeline is presented in Albee et al. (2021). This approach, including a target motion prediction functionality, a chaser motion planner, and a chaser tracking controller, will be tested in 2021 on the ISS with the ASTROBEEs.

#### Pre-Grasping Motion Planning

In deploying a SMS for target capture, a manipulator trajectory is needed to achieve the goal. Several secondary optimization goals, such as obstacle and singularities avoidance, fuel consumption, and base disturbance minimization can be sought, too. In most cases, the SMS is free-floating (full or partial mode), as described in *Dynamics of Space Robots in Orbit* and *Free-Floating Space Manipulator Systems*. In the pre-grasping phase, the manipulator arm moves from its home position to intercept a grapple fixture or point on the target at a rendezvous point with zero relative velocity.

Pre-grasping trajectory planning for robotic capturing of a tumbling satellite was presented in Aghili and Parsa (2008) without considering some operational requirements at the time of grasping. An EKF was incorporated in the robot planning to provide estimation of the target’s states and parameters needed for predicted motion trajectories (see *Vision-Based State/Inertia Parameter Estimation and Motion Prediction*), so that the robot’s end-effector could intercept the target’s grapple-fixture with zero relative velocity (to avoid impact). Capture of a satellite by a two degree-of-freedom manipulator using the Reaction Null Space method was presented in Piersigilli et al. (2010). In Aghili (2012), a predicted motion planning for the pre-grasping phase was presented that allowed minimizing a cost function consisting of a weighted linear sum of the travel time, the distance, the cosine of a line-of-sight angle (feasible alignment for robotic grasping), and a soft constraint on the acceleration limit. The pre-grasping trajectory planning and autonomous grasping of a tumbling using actual vision feedback were successfully demonstrated using a dual-arm robotic system used for simulating the motions of a tumbling satellite and a servicing space robot (Aghili, 2012). In Lampariello and Hirzinger (2013), a direct single shooting method was used to treat the grasping problem with inclusion of robot joint position and velocities constraints (to also account for dynamic singularity avoidance, see also *Singularity Avoidance*), as well as the chaser free-floating dynamics. Due to the long computation times involved in the motion planning, a look-up table approach was presented in Lampariello and Hirzinger (2013) to provide feasible optimal solutions for a range of spin rates of the target in a useful time, however with computation of the trajectories on a computer on ground. A reactionless approach of a two-arm space robot, in the pre-capture phase, where the motion of the second arm was used as a fuel-free means of attitude disturbance cancellation, was presented in James et al. (2016). In Flores-Abad et al. (2017) an optimal control problem was also formulated with the indirect method in joint space, aiming at minimization of torque applied by the robot on the free-floating chaser, while moving towards the grasping point. The problem was solved numerically, addressing the grasping task under the uncertainty of the initial and final positions of the robot end-effector. Yang et al. (2018) presented a multi-priority coordinated trajectory planning method for a dual-arm SMS to capture a target satellite. The method is based on a projection of the null-space of the generalized relative Jacobian matrix of the robotic system. They showed success of capturing a spinning (at 1°/s) satellite using simulation. In Lampariello et al. (2018) the planning method was extended to handle sensor-driven motion constraints and was validated on DLR’s OOS-SIM hardware-in-the-loop simulator, to allow for sensory-feedback trajectory tracking, with sensory-feedback throughout the complete task execution. In Aghili (2021) an adaptive and fault-tolerant vision-guided robotic system was developed for capturing a space object having drifting and tumbling motions subject to occlusion of the vision system. An optimal path planner brings the robot end-effector to the grasping point of a moving target as quickly as possible, subject to multiple constraints such as acceleration limit, smooth capture, and collision avoidance. Experimental results demonstrated smooth capture of a free-floating satellite mockup in spite of system uncertainties and a complete failure of the vision system due to occlusion. Other ideas resulting in reactionless (in terms of spacecraft attitude) manipulator path-planning, were the Reaction Null Space (Nenchev et al., 1996; Piersigilli et al., 2010) and the Zero Reaction Maneuver (Yoshida et al., 2001).

#### Post-Grasping Motion Planning

Having grasped the uncontrolled drifting and tumbling target, the space manipulator should gently exert force and torque to the target for stopping its drift and transferring its angular momentum to the servicing SMS. Several studies on optimal path planning for stabilization of a tumbling satellite in the post-grasping phase exist. A path planning method must consider the permissible bounds on the interaction moments between the SMS and its target during detumbling. Otherwise, excessive forces and moments may lead to mechanical damage or actuation saturation of the SMS attitude control system. Other constraints include robot workspace limits and joint velocity limits (especially due to dynamic singularities (Papadopoulos, 1993), see also *Singularity Avoidance*). The principle of conservation of momentum was first used by Dimitrov and Yoshida (2004) to damp out the chaser-target relative motion. An impedance control scheme for a free-floating space robot in grasping of a tumbling target with model uncertainty was presented by Abiko et al. (2006). These control schemes do not impose motion or force/torque constraints.

The problem of path planning and control of space manipulators to stabilize a tumbling satellite in the post-grasp phase was postulated and addressed in Aghili (2008). The development of fast detumbling maneuvers subject to torque restriction followed (Aghili, 2009). However, the coupling between dynamics of the rotational and translational systems was ignored and thus the planned trajectory was not truly optimal. In Lampariello and Hirzinger (2013) and Lampariello et al. (2018), the post-grasping task (rigidization) was again addressed with the direct single shooting method as an NLP, optimizing the mechanical energy of the robot arm. Particularly, in Lampariello et al. (2018), this phase was re-planned onboard, to account for tracking errors in the previous approach phase (see *Pre-Grasping Motion Planning*), while favoring the fulfillment of the position-dependent motion constraints (such as collision avoidance and robot manipulator workspace limits). It was in fact found that end-effector forces were well below the operational limits.

Other methods for post-capture control of tethered (the gripper is attached to a space platform through a tether) or articulated space-manipulators have been proposed (Nguyen-Huynh and Sharf, 2013; Wang et al., 2015; Huang et al., 2016; Zhang, et al., 2017; Wang et al., 2018; Huang, et al., 2019). Nguyen-Huynh et al. developed an adaptive reactionless motion and parameter identification in post-capture of a space object grasped by a manipulator (Nguyen-Huynh and Sharf, 2013). Wang et al. (2015) proposed a novel control scheme to realize stabilization of tumbling combinations after target capture by coordination of a tethered space manipulator and thrusters accommodated on the base of the space manipulator. A detumbling strategy and coordination control of kinematically redundant space robots after capturing a tumbling target was proposed by Wang et al. (2018). Chu and Wu (2018) presented a new self-learning soft-grasp control algorithm based on the variable stiffness technology for target capturing by a free-floating space manipulator. Self-collision avoidance and avoiding the collision with target by manipulator links might be another cost function parameters (Huang et al., 2019).

Optimal control strategies for the post-grasping phase, where the optimal controller damps out both translational and rotational motions collaboratively and simultaneously by taking advantage of the coupling between dynamics of translational and rotational systems was proposed in Aghili (2020). The optimal controller minimizes a cost function, which can be time, distance, or energy, while ensuring that the magnitude of the interaction force and torque between the manipulator’s end-effector and the satellite remain below their prescribed safe values. In Virgili-Llop and Romano (2019) the authors extended their work in Virgili-Llop et al. (2019) to solve the guidance task simultaneously for the capture and detumble maneuvers, consolidated by extensive numerical simulations and hardware-in-the-loop experiments. A dual-integral sliding mode planning method based on the reconfiguration of the reaction wheels is proposed in Hana et al. (2020) for the stabilization control problem of a combined spacecraft after multiple impacts during target capture. A non-holonomic path-planning technique based on a particle swarm optimization was proposed and applied to target berthing and target post-capture base re-orientation (Xu, et al., 2009).

#### Singularity Avoidance

Of relevance to the capture motion planning task is also singularity avoidance, which in the case of free-floating robot dynamics, is particularly challenging, since a given end-effector pose may be singular or not, depending on the path taken to reach it (see *Dynamics of Space Robots in Orbit*). Efforts have been dedicated to describing the location of the singularities in the robot workspace. The *Path Dependent* and *Path Independent Workspaces* were defined (Papadopoulos, 1992; Papadopoulos and Dubowsky, 1993); the latter was used to ensure dynamic singularity-free manipulator motions, while minimizing the SMS base disturbances. To plan manipulator trajectories for free-floating systems, a Lie algebra approach was employed for Cartesian motion planning (Papadopoulos, 1992). To avoid long duration trajectories, a Cartesian point to point path planning methodology using high order polynomials, was employed to specify the desired path directly in joint-space (Tortopidis and Papadopoulos, 2007). The accessibility of final configurations was extended drastically, while free parameters were determined by optimization techniques. Another approach was based on flatness theory extended to three-link spatial space robots (Xu et al., 2008). A similar approach was presented in Agrawal et al. (2009).

By making use of the inverted chain formulation (Abiko et al., 2006) and of free-floating robot dynamics properties presented in Cusumano et al. (2004), an efficient and complete method for generating singularity maps in the joint space of a 6-DoF free-floating robot is presented in Calzolari et al. (2020). Given the location of the singularities, the singularity avoidance can be treated as a collision avoidance problem, to increase the efficiency of the motion planning task in a nonlinear programming setting. Assuming that a Cartesian path is predefined, a method was proposed to yield initial system configurations that ensure that the predefined path avoids dynamic singularities (Nanos and Papadopoulos, 2012).

### On-Orbit Assembly

Different works in the literature conceptually discussed the on-orbit assembly of space telescopes. The Rendezvous and Docking assembly principle have different drawbacks, including high risk of collision, high requirement for the GNC system and large fuel consumption (She et al., 2020). To accomplish the task with space robots, the free-floating dynamics is typically omitted, since the parts to be assembled and the robotic arm which assembles them, are both hosted on the same spacecraft.

Examples of robotic assembly planning for this specific task can be found in She et al. (2020) and Martinez-Moritz et al. (2021). The motion planning task was divided in the latter into a global and a local layer. The global layer faces the challenge of first planning the assembly order, as well as of creating the instructions to be followed by the planner of the local layer. Methods to achieve this task solve the so-called robotic assembly planning problem (Martinez-Moritz, et al., 2021). The local layer consists of a constrained path planner that plans manipulation tasks to place single parts into the assembly. Different methodologies may be adopted here, including Rapidly-exploring Random Trees (RRT) - based methods, such as RRT-Connect (Kuffner and LaValle, 2000), Constrained Bi-directional Rapidly Exploring Random Tree (CBiRRT) (Berenson et al., 2009) for sampling-based path planners, CHOMP (Ratliff et al., 2009), STOMP (Kalakrishnan et al., 2011; Martinez-Moritz et al., 2021) and Genetic Algorithms (She et al., 2020) for optimization-based path planners.

## Feedback Control

### Control Schemes

Many control approaches can be referenced, depending on the task at hand. These include control for approaching a target, for capturing or interacting with a target, and for handling and servicing clients. Special mention must be made to robust control methodologies aiming to reduce the effects of uncertainties.

#### Control for Target Approach

Several works exist for the spacecraft velocity matching control, usually as a combination of planning and feedback control. An optimal control of a spacecraft approaching a tumbling target was developed in Xin and Pan (2011), minimizing the flexible motion induced by large angular maneuvers, using a nonlinear optimal control technique. In Buckner and Lampariello (2018) a tube-based *Model Predictive Control* (MPC) controller for tracking was implemented to track motion planning solutions for the chaser approach problem, see also Albee et al. (2021) for a mission pipeline related to these methods.

#### Capturing/Contacting a Target—Impedance/Compliance Control

The interaction of a SMS with its environment is important in many tasks and many research works focus on this challenge. Although some researchers have paid attention to the importance of contact control and performed various in-orbit contact dynamics analyses since the 90s’ (Ma, 1995), most control studies ignore the control of the contact force itself, as the contact dynamics is highly nonlinear and hard to model and control. Recently, hybrid impedance controllers for the capture and control of a rotating object by a free-floating space manipulator (Wu et al., 2017; Mou et al., 2018a) have been proposed.

In Yoshida et al. (2004), the concept of *impedance matching* was adapted to model the contact motion between a SMS and a non-cooperative target and studied whether contact with the target is maintained or lost. The *virtual mass* concept for using impedance control on-orbit has been proposed, aiming to represent the influence of the end-effector impedance on the target (Nakanishi et al., 2010). In Uyama et al. (2012), the impedance controller is considered in coordinates relative to the target, to reduce the dynamics of the contact problem to those of a damped oscillator. In Rodriguez Perez et al. (2018) a novel method for tuning an impedance control scheme was presented, which ensures post-impact velocity matching between the servicer and target satellites. A method for grasping a partially cooperative tumbling satellite with a free-floating robot, by implementing a tracking controller in Cartesian and in joint space, as well as an EKF for providing robustness and a tumbling satellite velocity estimate for feedforward control during grasping, was presented in Lampariello et al. (2018). To capture a target robustly without precise motion tracking and large force interaction, a novel gripper design in conjunction with the application of an impedance control law was proposed in Hirano et al. (2017). To minimize interaction forces between a robot manipulator and a satellite, while maintaining contact, an approach based on *direct force control* in the presence of a rigid grasp was proposed in Seweryn, et al. (2018), while a solution to minimize the risk of damage to the arm and thereby enhance contact performance was presented in Ma et al. (2015). However, both designs require control mode switching. For the docking of a SMS to a target with an impedance-controlled manipulator (Mitros et al., 2016; Mitros et al., 2017), the relationship of impedance gains to system parameters was established.

To allow for larger workspaces, free-flying SMSs have been considered. An extension of Hogan’s impedance control concept, the *Object Impedance Control* (OIC) has been developed for multiple robotic arms manipulating a common object (Schneider and Cannon Jr, 1992). To manipulate an object by a free-flying SMS with multiple arms on-orbit, the *Multiple Impedance Control* (MIC), which exploits the OIC, has been developed (Moosavian and Papadopoulos, 2010; Mitros et al., 2017). An Extended MIC method has been proposed for the dual-arm control of a passive object in space, in the presence of flexible appendages (Zarafshan and Moosavian, 2011). In a disturbance-based impedance controller, an end-effector desired trajectory generator provides the desired impedance behavior, while the desired motion is applied using a simple PD joint torque controller (Flores-Abad et al., 2018). Stolfi et al. (2017) extended the formulation of Impedance Control proposed in Nakanishi and Yoshida (2006) to a two-arm free-flying manipulator system with particular emphasis on the impact and post-impact phase with a target satellite. In Nagaoka et al. (2018) the detumbling and capture of space debris by a dual-arm space robot is accomplished by repeated impact, without precise estimation of the inertial characteristics and surface frictional roughness of a spinning rocket upper stage.

Based on the *passivity control method*, research, and experimental analysis on flexible joint manipulators with joint torque feedback has been developed in Ott et al. (2004) and Albu-Schaeffer et al. (2004). However, the nonlinear friction that exists at the joints was not explicitly addressed in these works. Thus, in practice these controllers were applied together with additional motor side friction compensation or disturbance observers. An impedance control with adaptive friction compensation for the dexterous robot hand has been proposed in Chen et al. (2011), implementing a friction EKF based observer, for adaptive impedance control of the fingers.

DLR took the lead in implementing the on-orbit impedance control experiment in ROKVISS and conducted an experimental study on joint parameters (Landzettel, et al., 2006). A compliant control mode (including force and impedance control) was tested in orbit with JAXA’s ETS-VII and showed good performance (Oda, 1999). The problem of detecting, isolating, and estimating the contact force for an orbital robot was addressed in Cavenago et al. (2021). A new observer was presented based on the dynamics in terms of the motion of the centroid of the whole robot and the joints, which was compared to the classical base-joint dynamics approach and validated with hardware on ground. The same method was extended in Cavenago et al. (2021) to include reaction wheels to thruster actuation of the base body, as well as a reaction control strategy, which aimed at avoiding the buildup of the contact force and possible instabilities.

The Special Purpose Dexterous Manipulator (SPDM) has been extensively utilized to handle various ORUs for ISS maintenance operations (Oshinowo et al., 2006). SPDM is a dual arm manipulator where each 7 degree-of-freedom arm is approximately 3.3 m long and is mounted on a single DoF body joint. It can complete human scale delicate servicing tasks with maximum tip velocities 7.5 cm/s-2.5°/s. In the teleoperation mode, SPDM is capable of positioning its end-effector relative to a target within 0.6 cm-2.0°, while in the automatic mode it is capable of adjusting the position and orientation of its end-effector in increments of 0.2 cm or 0.1° in any direction using Force-Moment Accommodation (FMA) control (Fulford, 1999; Aghili et al., 2001; Mukherji et al., 2001; Oshinowo et al., 2006). The capability of a space manipulator to effectively perform tasks involving contact hinges on the availability of an adequately accurate force-moment feedback. For many robots, force feedback is provided by a force-moment sensor (FMS) installed at a robot’s wrist. On-orbit calibration of SPDM force-moment sensors was studied in Aghili (2000).

#### Coordinated Control and Handling/Servicing Space Objects

When a SMS executes tasks on a serviced vehicle or a passive object (such as orbital assembly part or orbital debris), the control of both the manipulator configuration (to perform the required task) and the spacecraft attitude/position (to avoid collisions and loss of contact with the operations command center), is required. The coordinated control of the spacecraft and its mounted manipulators is an important control mode, which today tends to be addressed by a single controller.

The coordinated control of SMS in which both the spacecraft and its manipulators are controlled was developed employing a Transposed-Jacobian controller with inertial feedback (Papadopoulos and Dubowsky, 1991b). Coordinated control of a spacecraft attitude and its manipulator (partial free-floating mode) for OOS applications was presented in Aghili (2009). Α coordination controller for the combined system of a SMS and its target, considered as a manipulator payload, aimed at controlling the attitude of the target (Huang et al., 2016). A control strategy considering the servicing vehicle base and the manipulator as a single multi-body system subject to coordinated control was presented in Sabatini et al. (2017), with the goal of approaching and grasping a target spacecraft. In De Stefano et al. (2019), a coordinated control was presented for end-effector tracking and base regulation, while focusing on the effects due to the different sampling rates of the manipulator and base controllers, which can generate stability issues. The approach task to a tumbling target with a fully actuated free-flying robot was addressed in Mishra et al. (2020), where a cascade interconnection of a geometric EKF observer and a geometric controller were validated in simulation.

A coordinated control scheme which considers the contribution of reaction wheels to the system angular momentum, has been studied in Jayakody et al. (2016) modifying the *Adaptive Variable Structure Control* (AVSC) scheme to a SMS. In Antonello et al. (2019), coordinated control of both the servicing vehicle and the manipulator end-effector, in face of disturbances (e.g., point contact with a serviced satellite), was proposed. A method for coordinated control of both the manipulator end-effector and the servicing vehicle attitude, and the translation of the global system CoM, was proposed (Giordano et al., 2019). A control strategy which uses thrusters, reaction wheels, and robotic arm drives in a coordinated way to limit the use of the thrusters in both cases with and without contact is presented in Giordano et al. (2020). A unifying framework for whole-body control of orbital robots can be found in Giordano (2020), in which the advantages of common free-floating and free-flying strategies are merged, resulting in controllers that are more fuel efficient than the classical spacecraft positioning controllers.

To control multi-arm space robots in coordination with the spacecraft base, several schemes have been proposed, such as a model-based control algorithm (Moosavian and Papadopoulos, 1997), and an adaptation of the AVSC (Shi et al., 2017). The use of a second arm as a balancing mechanism, while the primary manipulator performs the desired task, was studied in Xu et al. (2017).

A compliance/impedance controller for the end-effector, integrated as part of a coordinated control scheme can both stabilize the servicing vehicle, and control the manipulator end-effector. A coordinated control method for a single manipulator capturing of a tumbling target, implementing a fast, on-line updating manipulator path planner and end-effector compliance control, was proposed (Gangapersaud et al., 2019). Coordinated detumbling of a non-cooperative captured target, with simultaneous servicing vehicle attitude PD control, was developed in Hirano et al. (2018). However, both abovementioned methods do not consider singularity avoidance or manipulator workspace constraints.

Besides the coordinated control schemes, other controllers have been proposed for the captured target handling task, such as a control method for handling captured passive objects, aiming at reduction of flexibility-induced vibrations (Dubowsky and Boning, 2007), or methods proposed to maintain firm grasp (Hiramatsu and Fitz-Coy, 2007). The handling of a passive object by multiple space robots, was studied in Rekleitis and Papadopoulos (2015), proposing a hybrid control scheme with on-off thruster control of the SMSs, while their manipulators could apply continuous forces on the passive object.

#### Control in the Presence of Parametric Uncertainties

Two main approaches exist in treating parametric uncertainty: robustness and adaptation. The nonlinear robustness and parameter sensitivity field is rather limited, with most works relying on special features to prove stability under uncertainties. Nonlinear *Sliding Mode Control* (SMC) (Slotine and Li, 1991) can be used, but it suffers from drawbacks such as excessive control effort (Dastidar, 2010), and state oscillation around the desired values; the later can be mitigated using higher order SMC (Ferrara and Incremona, 2015). Linearization, when applicable, can be employed to allow use of linear robustness tools Rekleitis and Papadopoulos (2014). The problem of tracking control with a guaranteed performance for free-floating SMS with uncertainties and external disturbances, was studied using an adaptive nonlinear $H_{\infty }$ controller via neural networks (Taveira et al., 2006). A non-linear $H_{\infty }$ controller has been proposed for a SMS operating in a partial free-floating mode (Seddaoui and Saaj, 2019). However, the design of nonlinear $H_{\infty }$ controllers is more complex than the design of linear ones since the design variables are not directly related to system performance.

In the adaptation approach, controller parameters are adapted so that the desired response is obtained despite parameter variations (Slotine and Li, 1991). However, they are subject to limitations, especially in free-floating systems, in which classical adaptive control laws are not applicable readily. Thus, while adaptive control has been proposed for free-flying robotic systems (e.g., Ulrich et al., 2016), its use in free-floating ones is restricted. Adaptive control has been proposed for free-floating robotic systems handling a captured passive target, either using the base reaction to dampen vibrations (Abiko and Yoshida, 2010), or generating reactionless manipulator motions not disturbing the spacecraft attitude (Nguyen-Huynh and Sharf, 2013). A task-space adaptive controller has been proposed in Wang et al. (2017), however it requires four adaptation laws simultaneously, and an online solution of a differential equation.

Parameter identification methods can be used to estimate accurately system parameters, and concurrently be used in any stable non-linear controller. Methods for concurrent parameter identification and adaptive control have been proposed for a simplified point-mass system (Espinoza and Roascio, 2017) or for a full space robot that assumes only the last manipulator link (including the captured target) as unknown, while it also requires noisy acceleration measurements (Zong et al., 2019). In Christidi-Loumpasefski et al. (2020) a fast, and reliable parameter identification method previously developed by the authors, was further enhanced, to identify all required parameters for the complete system dynamics reconstruction in Cartesian and joint space and provide on-the-fly accurate parameter estimation for control, resulting in a Self-Tuning Controller (Slotine and Li, 1991).

### Visual Servoing

Visual servoing approaches for manipulation of space objects in complex scenarios and automated rendezvous and docking of non-spinning spacecraft have been proposed for various missions (Wertz and Bell, 2003; Ruth and Tracy, 2004; Evans III and Mulder, 2006). Wertz and Bell (2003) gave an overview of hardware and software technologies (sensors and actuators) required for autonomous rendezvous and docking of two spacecraft started at a remote distance. The terminal phase of the *Demonstration of Autonomous Rendezvous Technology* (DART) mission that includes proximity maneuvers for rendezvous to a cooperative spacecraft under an advanced video guidance sensor is described in Ruth and Tracy (2004).

Adaptive control law for spacecraft rendezvous and docking under measurement uncertainty such as aggregation of sensor calibration parameter, systematic bias, or some stochastic disturbances was proposed in Singla and Junkins (2006) and Aghili and Su (2016). The development and experimental validation of adaptive visual servoing for on-orbit servicing was presented in Aghili (2012); see Figure 3. The vision guidance problem for the shortest time was cast into the optimal control framework pertaining to two sequentially occurring maneuvers in the pre-grasping and post-capturing phases (Aghili, 2013). Adaptive deliberate planning was accomplished by combining a 3D registration algorithm and a constrained estimator allowing real-time estimation of required parameters and states (Aghili and Parsa, 2007). This integrated estimation and control architecture also allows fault detection and recovery of the visual feedback whenever the vision sensor generates erroneous information, i.e., caused by partial or full obstruction vision (Aghili et al., 2011). A visual servoing method for the approach, capture and rigidization of a tumbling target with a free-floating robot was presented in Lampariello et al. (2018). The visual servo was fed a desired command from a combination of a reference trajectory and the output of an EKF. The method was tested on an experimental facility on ground. A fault-tolerant and adaptive visual servoing for capturing free-floaters has been recently presented in Aghili (2021) that allows to choose the most appropriate control action in the face of environmental uncertainties or short-term failure of the vision. Experimental results demonstrated smooth capturing of a free-floating object in the present of partial or complete failure of the vision system.

### Telepresence and Teleoperation

Although impressive progress has been made in recent years with respect to the robotic automation level of non-trivial tasks, it is of key importance, especially in the space robotics environment, to be able to react quickly to unforeseen situations or to incorporate the integration of “human” intelligence from the beginning when performing robotic activities in space. To this end, telerobotics is the appropriate paradigm combining robotic (manipulation) and human capabilities (intelligence, strategy, problem solving). In this context, the term telerobotics subsumes the areas of *teleoperation* up to *telepresence*. Teleoperation covers the entire range of task execution at a remote location, including the use of intelligent autonomous systems. Telepresence, on the other hand, stands for the possibility of being quasi-immersively present at the remote location as an operator through a robotic avatar. To this end, experiments were carried out in and with the ISS in recent years to cover the entire range of telerobotics. However, it is worth pointing out that these experiments involved interactions with quasi-static environments. The effects of communication time delay and dropouts, of limited bandwidth, as well as of the operator’s misperception and limited field of view, make the execution of tasks on dynamic environments with telerobotic methods, such as the capture of a tumbling target, still challenging today. The ability of an operator’s fast response to a contingency, may be combined with an autonomous capture system in the context of shared control. However, this is a challenge that has not been studied adequately yet.

DLR’s and Roscosmos’s ROKVISS and KONTUR-2 experiments led the way to study the feasibility of employing robots as haptically coupled avatars for the user in both directions between Earth and orbit (Hirzinger et al., 2005; Artigas et al., 2016; KONTUR-2: Force-feedback Teleoperation from the International Space Station, 2016). METERON (Multi-Purpose End-To-End Robotic Operation Network), led/spearheaded by ESA, with partners NASA, Roscosmos, and DLR, conducted a suite of experiments to validate advanced technologies for space robotics operation and telemanipulation. Several robotic assets on Earth, including Rollin’ Justin (DLR), were controlled from on board the ISS using various command modalities. In contrast to KONTUR-2, the METERON SUPVIS Justin experiment placed an intelligent robot co-worker in the scenario of a planetary surface habitat for supervised autonomy teleoperation (Lii et al., 2015; Lii et al., 2018; Schmaus et al., 2018). The experiment aimed to demonstrate how robots, despite significant communication times, can be commanded to solve complex tasks. The local intelligence of the robot was used to implement the commands of an astronaut. However, the technology demonstrated in this scenario can well be applied for the case in which the robot is in orbit and the operator is on ground. NASA’s Robonaut I and II were designed for a wide variety of intra-vehicular activities on the ISS (Ambrose et al., 2000; Diftler et al., 2011). The robot can be teleoperated by coupling its stereo vision and dexterous capability with the user through augmented reality and finger tracking (Bibby and Necessary, 2008; Peters et al., 2003).

NASA, with the support of CSA, has started a Robotic Refueling Mission (RRM) on ISS using CSA’s SPDM (Gefke et al., 2017). RRM is a series of multi-phased ISS payload experiments designed to test and mature the tools and technologies associated with on-orbit robotic fueling services. The hardware is a 1.1 m × 1.1 m × 0.8 m module consisting of four robotic servicing tools, several tool adapters, a fluid (ethanol) transfer system, and multiple task boards, valves, and spacecraft blanketing representative of those found on existing satellites. The tools contained within RRM were actuated and controlled via SPDM, operated at ISS mission control with NASA personnel supporting on-orbit operations remotely from the NASA Goddard Space Flight Center. The primary robotic control method is remote high-level teleoperation with local closed-loop force-moment accommodation (thus reducing contact risk). Phases 1 and 2 of the mission have been successfully completed in 2013 and 2015, respectively. Phase 3 is focusing on technologies needed to transfer and long-term (<3 months) store super-cold cryogenic fluids (NASA, 2021).

## Ground Testbed Facilities

Ground testbed facilities have been used for spacecraft control hardware/software verification since various space programs began half a century ago (Schwartz et al., 2003). Due to the high cost of launch and operations associated with on-orbit repair, a spacecraft must operate reliably once it is placed in orbit. Therefore, realistic testing of spacecraft prior to launch, ideally with all hardware/software in place at system level, ought to be undertaken to ensure that the spacecraft functions as intended. One of the challenges of this approach is that testing must take place in a 1-g environment, whereas the actual system will eventually operate in a zero-g environment. This has motivated the building of testbed facilities in various government and university laboratories for the testing and verification of space robotic systems (Wilde et al., 2019).

### Zero-G Simulation of Free-Floating Space Objects

Since space manipulators are designed to work in a microgravity environment, they should also be tested in a microgravity environment. There are many technologies available to address the problem of reproducing the microgravity space environment, such as air bearings, cable suspension, neutral buoyancy, free-fall, magnetic suspension, large rotating wheels, and HIL simulation (Flores-abad et al., 2014a). However, of all of these, the air bearings are the most popular in industry for testing spacecraft.

Cable suspension achieves single dimensional weight compensation only. Adding a 3D gimbal at the end of the cable can simulate weightless rotation but it is an unstable emulation because the CoM of the simulated object must always align perfectly with the gimbal center, which is obviously not suitable for robot testing. Neutral buoyancy facilities, i.e., water tanks, have been used extensively for astronaut training. However, a functional spacecraft cannot be submerged in the water; in addition, viscous damping does not allow a space-representative dynamic environment and the water media also significantly affects sensor performance. A free-fall test through flying parabolas in aircraft can achieve zero-g in a 3-D environment but only for less than 30 s of testing time, not long enough for most space robotic operations. Magnetic suspension systems provide only a low force-torque dynamic environment with a small range of motion.

Air-bearing tables (Yoshida, 2003; Papadopoulos et al., 2008; Rybus and Seweryn, 2016) and spherical air-bearings (Schwartz et al., 2003) are commonly used for ground-based testbeds for testing the translation and attitude control systems of a spacecraft. An emulation of zero-g translational motion can be achieved by an air-bearing table on which a spacecraft translates on a flat surface perpendicular to the gravity direction while being floated on a cushion of compressed air with almost no resistance. This technique has been used for testing various space systems such as formation flying (Choset and Kortenkamp, 1999), free-flying space robots (Yoshida, 2003), orbital rendezvous and docking (Matunaga et al., 2000; Aghili and Su, 2016), capturing mechanisms of spacecraft (Kawamoto et al., 2001), and free-flying inspection vehicles (Choset and Kortenkamp, 1999).

Although an air-bearing table system can be utilized to test some physical components of spacecraft control systems, including the sensors and actuators, this system is limited to a two-dimensional planar environment. Spherical air-bearings have been used for spacecraft attitude determination and control hardware/software verification for many years (Schwartz et al., 2003). The earliest development and design of a satellite simulator based on spherical air-bearing with three axes of rotation has evolved into modern testbed facilities (Colebanket al., 1999; Schwartz et al., 2003). A spherical air-bearing yields minimum friction and hence offers a nearly torque-free environment if the CoM is coincident with the bearing’s center of rotation. The main problem with the spherical air bearing is the limited range of motion (within ±45° about horizontal axes) resulting from equipment being affixed to the bearing (Peck et al., 2003). Also, spherical air-bearings are not useful for simulating spacecraft having flexible appendages, because the location of the center-of-mass of such spacecraft is not fixed. Spherical bearings cannot be used to simulate a robot’s weightless rotation either because the mass center of a robot changes when the robot moves.

Although one can envisage combining the two air-bearing technologies in a testbed for reproducing both the rotational and translational motions (Tsiotras, 2014; Saulnier, et al., 2014), having a spacecraft mounted manipulator and complete freedom in all six rigid degrees-of-freedom is still technically difficult to achieve (Schwartz et al., 2003). Air-bearing supported testing of a large space manipulator requires a large and massive test mechanism to support the manipulator and the payload it handles. Since the supporting mechanism must move with its supported manipulator, it will alter the dynamics of the tested manipulator. Yao et al. (2018) recently developed a method to eliminate this unwanted side effect, so that the true dynamics of the tested manipulator can be understood.

Most motion testing systems allow the incorporation of real sensors of a satellite such as gyros and star trackers in HIL simulation loops. However, actuators such as reaction wheels or gas-jet thrusters have been simulated. The main idea in HIL simulation is to combine digital simulation of the robotic arm and physical testing of some of its hardware in the same framework. In other words, under the HIL simulation framework some of the SMS components are simulated by digital models and other components are represented by real physical hardware. Such an approach can take advantage of both digital simulation (for difficult-to-test items such as the long-reach arm in 3D zero-g space) and hardware testing (for difficult-to-model items such as contact dynamics). Rather than testing the control algorithm on a purely mathematical model of the robotic system, one can use real hardware in the simulation loop (Aghili et al., 2006; Aghili and Namvar, 2009). This allows key hardware to be physically tested along with the entire SMS system in operation, which is otherwise impossible in the 1-g environment. It also allows detailed measurement for accurate performance assessment of the system under the test.

**FIGURE 4:**
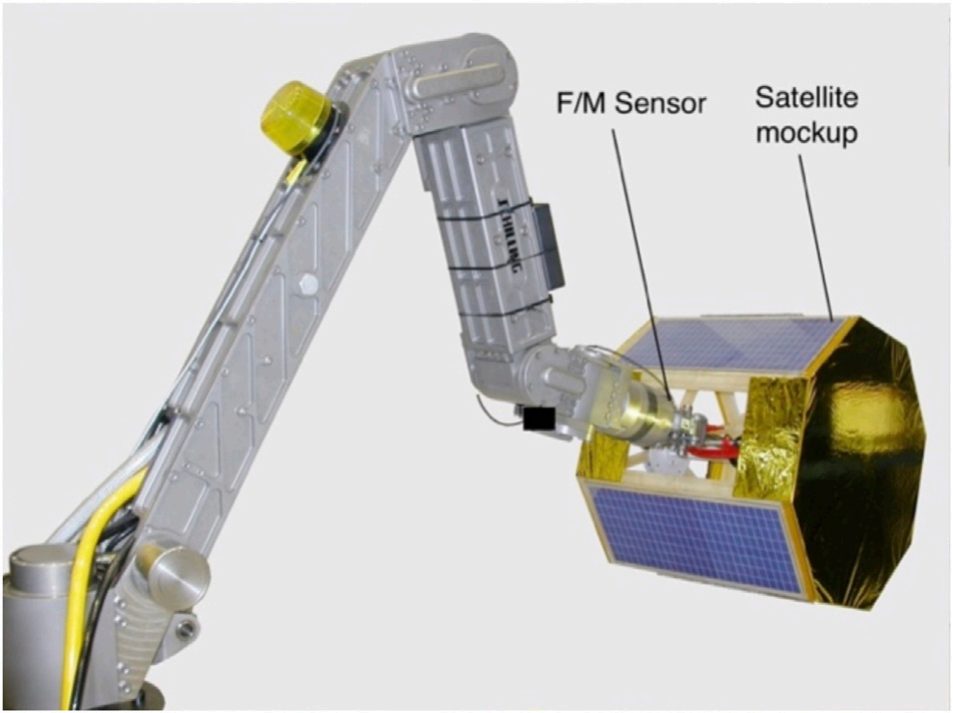
Zero-G satellite simulator (CSA).

The concept of the HIL methodology has also been utilized for design and implementation of various laboratory testbeds to study the dynamic coupling between a space-manipulator and its host spacecraft operating in free space (Dubowsky et al., 1994; Tarao et al., 2000; Yoshida, 2003). A system called the Vehicle Emulation System Model II (VES II) permits the experimental evaluation of planning and control algorithm for mobile terrestrial and space robot systems by using the so-called “admittance control” (Dubowsky et al., 1994). Similar concepts have been also pursued by other space agencies such as, DLR (Krenn and Schaefer, 1999), CSA (Aghili and Piedboeuf, 2000), and NASA (Ananthakrishnan et al., 1996) for different applications. A method to control a manipulator system grasping a rigid-body payload so that the motion of the combined system in consequence of external applied forces to be the same as another free-floating rigid-body (with different inertial properties) was developed in (Aghili and Namvar, 2009). This allows zero-g emulation of a free-floating space object under the test in a 1-g laboratory environment as shown in Figure 4. The controller consisting of motion feedback and force/moment feedback adjusts the motion of the test spacecraft to match that of the flight spacecraft, even if the latter has flexible appendages (such as solar panels) and the former is rigid. Satellite simulator testbed facility based on hardware-in-loop simulation technology to investigate capturing free-floating satellite under 1-g laboratory environment was developed at CSA (Aghili et al., 2008; Aghili and Parsa, 2007; Aghili, 2012). A dual-arm robotic system was utilized at the CSA ground testbed facility for replicating the motion dynamics of a servicer robot and a target satellite; see Figure 3. Ground simulations using hardware in-the-loop simulation to simulate the servicing arm and a parallel motion-based platform to replicate the client satellite has been developed also at NASA’s Goddard Space Flight Center (Carignan et al., 2014). A later and more general overview of the ground test facilities at the same NASA center is given in Roberts (2017). This robotic testbed platform was used to investigate the dynamic interaction between the servicing spacecraft and client satellite (Strube et al., 2012; Brannan and Carignan, 2013; Brannan and Carignan, 2017; Brannan et al., 2018; Brannan et al., 2020).

### Space Mechatronics Testbeds

Joint servomechanisms consisting of actuators, sensors, and controllers are among the fundamentals in mechatronics and robotics. Development of any new joint prototype ought to undergo extensive mechanical, electrical, and thermal tests at different stages of the development to make sure that the system works as intended. In robotics applications, these tests can be performed using a robot prototype built on developed joints. However, building a complete prototype of the robotic system is an expensive and inflexible process. Moreover, due to the iterative nature of the design process, the need for multiple robot prototypes makes it even more costly and time consuming. The challenge of testing space manipulators is even greater because they must be tested and validated in a 1-g laboratory environment whereas the actual robotic system will eventually work in an environment with different gravity, temperature, and ambient pressure.

Alternatively, testing of actuators and join prototypes can be carried out by mounting them on a dynamometer. Industrial dynamometers tend to use a flywheel and/or mechanical brake for loading the actuator. However, such a simple load does not represent a real manipulator. Hence, the extent of the test result is limited, and the real performance remains largely unknown until the actual robot becomes operational. Mechatronic testbeds that use dynamometers for testing vehicle dynamics and control have been developed based on the concept of HIL simulation (Brennan and Alleyne, 2000; Tartt and Moskwa, 2001). These test methods are limited to emulating loads with linear dynamics.

**FIGURE 5:**
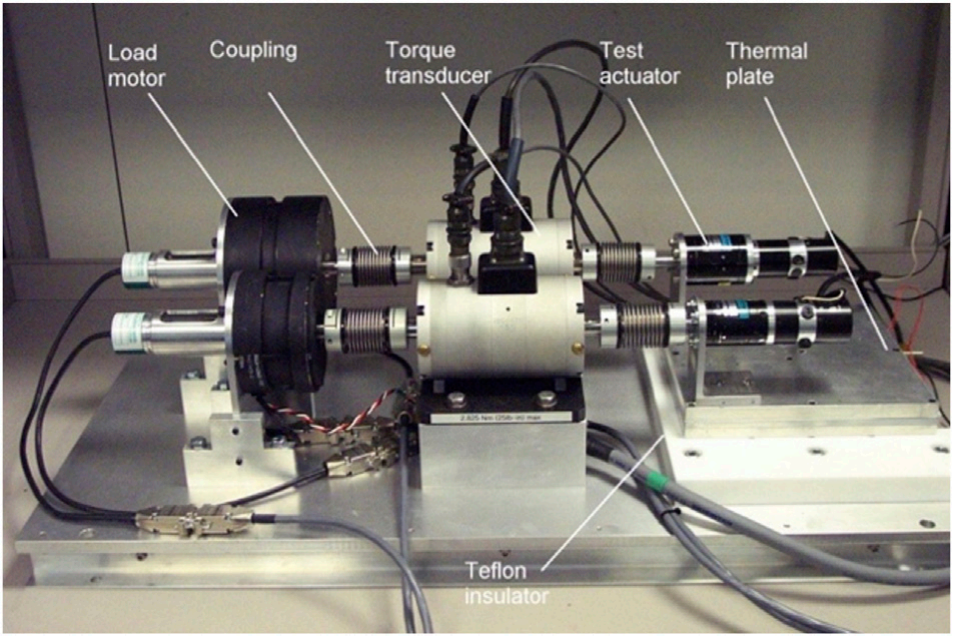
Testing the joints of a space manipulator on a dynamometer using actively controlled loads (CSA).

Design and development of a testbed facility for testing a range of actuators, used for either space robots were first reported in Aghili (2005, 2006). Unlike industrial dynamometers that apply constant braking torques, or vehicle dynamometers that are capable of emulating linear loads, a dynamometer utilizing active loads that generate loading torques corresponding to a prescribed mode was employed, see Figure 5. High fidelity joint torque emulation is made possible by incorporating the measurement of joint torque as well as joint angles and velocities in a composite feedforward/feedback loop. The role of the feedback control is to modify the simple inertia of the rotors of the load motors to match the nonlinear and coupled dynamics of manipulator links. Such a testbed system allows testing the complete joint prototypes of a manipulator without needing to construct the manipulator. This can reduce significantly the cost associated with the development of space robots, so that the joint prototypes can undergo test under a space-like thermal/vacuum environment.

### Space Manipulator Task Verification Facilities

Space robots have become viable means to perform complex extra-vehicular robotic tasks as they have proven to play critical role in construction and maintenance of the ISS. Assembly of the ISS was not possible without the iconic Canadarm2, while the SPDM has been extensively utilized to handle various ORUs for ISS maintenance. Station construction and maintenance operations mostly involve robotic contact tasks and therefore they must be first carefully planned and then properly controlled to avoid wedging, jamming, or overloading during the insertion or removal operation of ORUs. The verification of large space robots on the ground is challenging as these long-reach and lightweight robots are designed to work only in a microgravity environment and thus they cannot do real 3D operations on ground.

**FIGURE 6:**
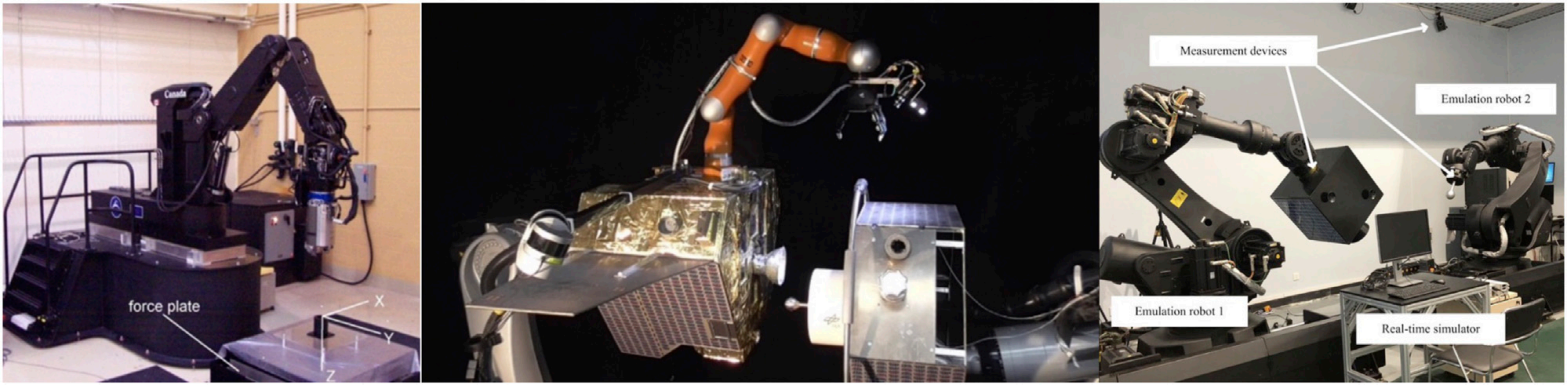
**(A)** SPDM Task Verification Facility (CSA), **(B)** Capturing task at the OOS-SIM facility (DLR). **(C)** Manipulator Test and Verification Facility (MTVF) developed by China Academy of Space Technology (Liu, et al., 2018).

Space agencies have built sophisticated testbed facilities for verification and validation of on-orbit contact tasks through implementation of HIL simulation technology using robotic manipulators capable of operating in 1-G laboratory environment. The earliest HIL simulation facility was built to investigate the berthing of a Space Shuttle onto ISS by the Shuttle Remote Manipulator System (Canadarm) (Tobbe et al., 1991). It followed by development of the SPDM Task Verification Facility (STVF) at CSA to verify dexterous tasks to be performed by SPDM on the ISS (Aghili and Piedboeuf, 2000; Ma et al., 2004; Aghili and Piedboeuf, 2006; Aghili, 2019); see Figure 6A. Similar HIL simulator facility to simulate on-orbit servicing robots performing contact tasks have been built at DLR (Krenn and Schaefer, 1999; Ma et al., 2012; Artigas et al., 2015). Bandwidth limitations of HIL simulator involving contact tasks regarding contact stability and fidelity performances have been the subject of several studies (Krenn and Schaefer, 1999; Aghili and Piedboeuf, 2006).

More recently, a passivity-based approach for simulating satellite dynamics on a position-controlled robot equipped with a force–torque sensor was presented in De Stefano et al. (2019). Time delay and discrete-time integration effects were analyzed from an energetic perspective and compensated through a passivity-based control strategy to ensure a faithful and stable dynamic simulation with position-controlled robots. The benefits of the proposed strategy were demonstrated on the OOS-SIM, shown in Figure 6B. An exhaustive analysis of energy-based control for simulation of multi-body dynamics using robotic facilities was presented in De Stefano and Marco (2018). The first example of a prolonged contact between the two industrial robots in the OOS-SIM, which results from the loop closure with the torque-controlled Light-Weight Robot (LWR, orange robot Figure 6B) when simulating capture, was presented in Lampariello et al. (2018). A stable prolonged contact was possible thanks to the sufficiently compliant behavior of the LWR, although some periodic disturbances were visible in the equilibrium regulation point, to be attributed to the intrinsic time delay in the simulator.

Since SPDM could not be directly tested for 3D contact operations on ground, after several trade-off studies, a ground-based HIL simulation facility consisting of an SPDM real-time dynamic simulator, a hydraulic manipulator (for mimicking SPDM dynamic behavior), and the real SPDM end-effector and payload mockups was developed at the Canadian Space Agency (CSA) (Aghili and Piedboeuf, 2000; Aghili and Piedboeuf, 2006; Aghili, 2019) for high-fidelity task verification of the SPDM. Since the simulating robot interacts with a physical environment, contact dynamics modeling is not required and hence many technical difficulties associated with contact dynamics modeling are avoided; see Figure 6A. On the other hand, the hydraulic simulating manipulator is not dynamically and even kinematically equivalent to the reference SPDM robot. Therefore, the greatest challenge in a high-fidelity simulating robotic system is to maintain dynamical similarity between the simulating robot and reference robot through proper control architecture. It turns out that this goal can be achieved though closed-loop impedance matching of the two robots so that the ground-based simulating manipulator can generate contact forces and transitional impact which closely match those expected from SPDM during on-orbit operations (Ma et al., 2004; Aghili and Piedboeuf, 2000; Aghili, 2019).

Another challenge for the emulating robot is the uncertainty associated with the environment impedance, which affects the contact stability and fidelity performance of the simulating robot. In essence, these challenges can be addressed by a systematic robust control approach to find the best compromise between fidelity performance and contact stability given varied range of environment impedances and limited bandwidth of the simulating robot. A robust impedance-matching of manipulators is presented in Aghili (2019) to generate high-fidelity contact force profiles in consequence of either operator commands or impulsive force caused by pre-impact velocity to match those of a space robot as closely as possible.

The idea of shaping the dynamics of the emulating manipulator to represent a scenario of interest was also applied in Mishra et al. (2020), where a fixed-based manipulator was used to simulate an orbital robot. The Lagrange-Poincare equations were used to describe the orbital robot’s dynamics, which reveal a block-diagonalized inertia, such that noisy joint acceleration/torque measurements were avoided in the computation of the spacecraft motion due to manipulator interaction, even while considering external forces. The chief advantage of this method is physical consistency of the simulation. The effectiveness of this approach was validated through DLR’s OOS-SIM hardware-in-the-loop simulator of a fully actuated orbital robot, while interacting with the environment. The dynamic shaping idea was also applied in De Stefano et al. (2021), where the OOS-SIM facility was used to simulate the relative motion between a very large tumbling target and a manipulator-equipped spacecraft. By exploiting a Lagrangian matching relative to a nominal motion, the simulated dynamics replicated by the robots enables motions of large satellites to be reproduced. The benefit of the method was demonstrated through experiments on the OOS-SIM facility for the grasping of ENVISAT, a free-tumbling satellite and the largest space debris in Low-Earth-Orbit (see also *Other General Software and Hardware Technology Developments*).

To support the research and development of the Chinese Space Station Program, an HIL Space *Manipulator Test and Verification Facility* (MTVF) was developed by the China Academy of Space Technology (Mou et al., 2018; Liu et al., 2018). As shown in Figure 6C, the system consists of two large industrial robots, one physically simulating the 3D motion of the end-effector of a space manipulator or its grasped payload, and the other simulating the payload to be grasped or the worksite the grasped payload will be in contact with Ding et al. (2021). Each of the customer-built industrial robots can handle 300 kg payload. Each industrial robot can translate for a short distance on a rail on the base.

The system used the HIL concept of combining digital simulation of an in-orbit space manipulator and a hardware testbed of real contact hardware. Such an industrial-robot based HIL simulation concept has been widely used in space industry (Flores-abad et al., 2014a). However, the uniqueness of the MTVF is to use custom built industrial robots to achieve better HIL simulation performance with a few special measures: a) it implemented 1,000 Hz commanding cycle in both joint and end-effector control loops for more stable HIL dynamic response; b) it increased gear ratio for smaller maximum speed but higher accelerating capability; c) it had an end-effector force/moment control capability; d) it used a dynamics-model based feedforward loop to reduce nonlinear effects of the industrial robots. These enhanced capabilities are not readily available from commercially-off-the-shelf industrial robots, but they are essential to help achieve stable and accurate HIL simulation of the impact-contact behavior of a space manipulator. Just as all the other existing major HIL simulation facilities, this system also employed the strategy of 6-DoF impedance match in operational space to ensure the fidelity of the HIL contact simulation results.

## Missions and Technology

Although proper orbital capture and manipulation missions are still scarce, quite a few studies were carried out in recent years on these topics and many new missions are in plan. The robotic technology related to these missions is also developing at an always increasing pace, as described next.

### Missions and Mission Studies

#### ESA’s and DLR’s OOS Mission Studies

**FIGURE 7:**
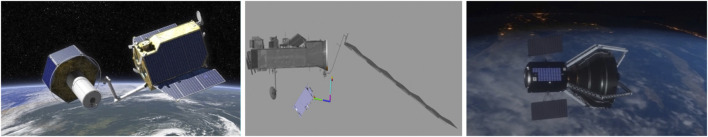
**(A)** DEOS (DLR), **(B)** e.Deorbit (ESA), **(C)** ClearSpace-1 (ESA).

After the pioneering ETS-VII and Orbital Express missions in 1998 and 2007, respectively (Yoshida et al., 2016), in which a cooperative, attitude-controlled target satellite was captured by a free-flying robot, some mission studies focused on the capture of a non-cooperative tumbling target. In DLR’s DEOS study (Reintsema et al., 2010), the mission goal was to capture a small tumbling satellite with a free-floating robot, in both the autonomous and tele-presence operational modes (the latter through an operator on ground). The e.Deorbit study was about an Active Debris Removal mission, promoted by the Clean Space initiative (Clean Space, 2012), given that the ENVISAT was going to be deorbited into the Earth atmosphere (e.deorbit Study Team, 2015). With a length of 26 m and a mass of 8 t, the chaser required to synchronize its motion with the Grasping Point on the target during capture. The two scenarios are shown in Figures 7A,B. In both studies, a torque-controlled kinematically-redundant robotic arm, based on DLR’s robot hardware technology (see *Arms, Grippers, and End-Effectors*), was used to provide compliant behavior at contact. Details of the e.Deorbit study were provided in Jaekel et al. (2018). Following the latter study, an Airbus DS-led e.Deorbit Consolidation Phase Study was carried out, based on the Airbus Spacetug and an MDA manipulator, in which the capture is preceded by a contactless detumbling maneuver (Estable et al., 2020). In the same spirit, ESA is currently financing the ClearSpace-1 mission (Figure 7C), with a caging capturing concept, consisting of multiple arms wrapping around the target. The latter is intended in this case to be the upper stage of a Vega rocket, with 100 kg of mass.

A different line of development for capturing a satellite for servicing, is the one first developed in the study *Orbital Life Extension Vehicle* (Smart-OLEV) (Kaiser et al., 2008). Here, the apogee motor of geostationary satellites is used as a docking port for a chaser, equipped with a dedicated docking mechanism. The chaser is then used to provide extra capacity for orbital and attitude control of the target, thus extending the latter’s operational life. The same idea was used ten years later in the two *Mission Extension Vehicle* (MEV) missions and in the *Mission Robot Vehicle* (MRV) concept (see *Northrop Grumman’s MEV and MRV*).

#### DARPA’s Robotic Servicing of Geosynchronous Satellites (RSGS)

**FIGURE 8:**
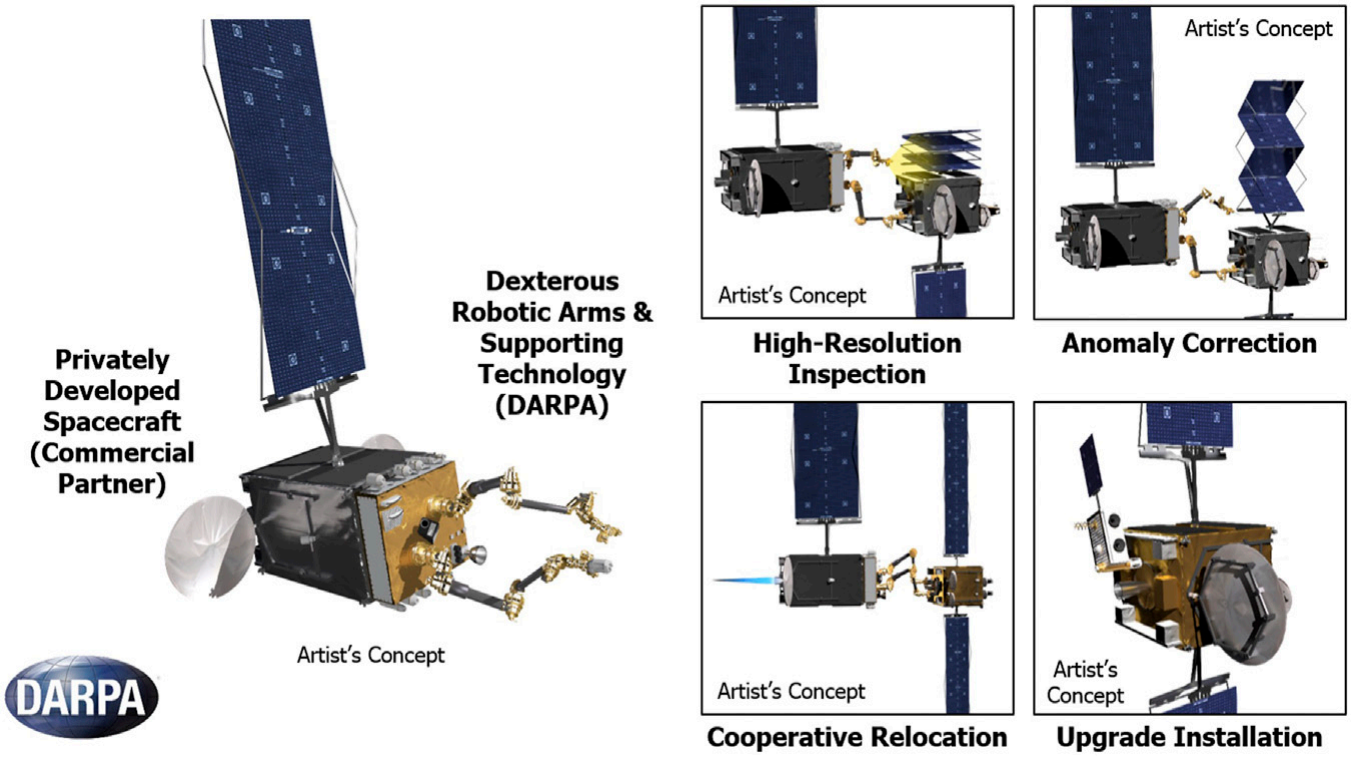
Robotic servicing vehicle (RSV) and envisioned missions (Parrish, 2021).

RSGS will be DARPA’s second OOS technology demonstration mission after the Orbital Express mission which was launched in 2007 and successfully demonstrated on-orbit robotic fuel transfer and capture of a cooperative client satellite (launched together with the servicing craft). Unlike Orbital Express and all the other prior OOS missions flown so far, RSGS will directly serve a client satellite in a GEO orbit. The mission (Figure 8) intents to (Parrish, n.d.):

1) demonstrate in or near GEO that a robotic servicing vehicle can perform safe, reliable, useful, and efficient operations, with the flexibility to adapt to a variety of on-orbit missions and conditions;
2) demonstrate satellite servicing mission operations on operational GEO satellites in collaboration with commercial and United States Government spacecraft operators;
3) support the development of a servicer spacecraft with sufficient propellant and payload robustness to enable dozens of missions over several years.

Originally DARPA teamed with Maxar Technologies for developing the RSGS mission. It recently changed the partnership to Northrop Grumman/ATK to integrate the RSGS into a new Northrop Grumman mission called Mission Robotic Vehicle (MRV).

#### NASA/SSL OSAM-1 (Formerly Restore-L)

**FIGURE 9:**
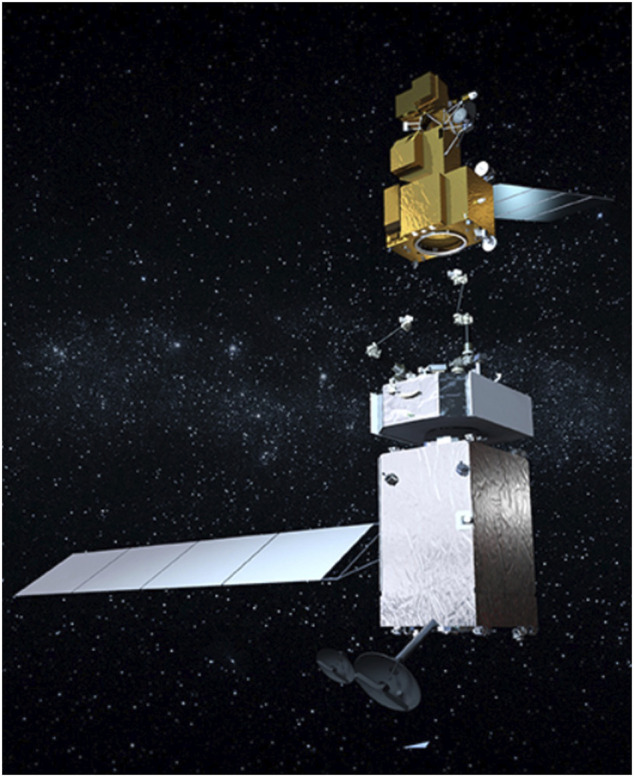
NASA/SSL OSAM-1 mission (NASA/GSFC, 2021).

According to (NASA/GSFC, n.d.), OSAM-1 mission is planned to perform an autonomous rendezvous with Landsat-7 in low Earth orbit (LEO) followed by refueling and orbit relocation. This endeavor requires two robotic arms and the development of a reliable propellant-transfer system (Figure 9). The Landsat-7 is an unprepared client, not originally designed with on-orbit servicing in mind, and its functional lifespan will be lengthened by this servicing mission. The OSAM-1 spacecraft will also include another payload called Space Infrastructure Dexterous Robot (SPIDER).

SPIDER includes a lightweight 5-m robotic arm, bringing the total number of robotic arms flying on OSAM-1 mission to three. SPIDER will assemble seven elements to form a functional 3-m communications antenna and manufacture a 10-m lightweight composite beam. The robotically assembled antenna will demonstrate Ka-band transmission with a ground station. SPIDER operations will help mature space technologies with many potential cross-cutting applications, including (Shoemaker et al., 2020):

1) enabling new architectures and capabilities for a wide range of government and commercial missions;
2) enabling In-space construction of large communications antennae and telescopes;
3) eliminating volume limits imposed by rockets;
4) replacing some astronaut extravehicular activity tasks with precision robotics; and
5) introducing the potential for longer mission durations enabled by planned or unplanned maintenance.

The mission was originally scheduled to be launched in mid-2020s (Henry, 2020).

#### Northrop Grumman’s MEV and MRV

**FIGURE 10:**
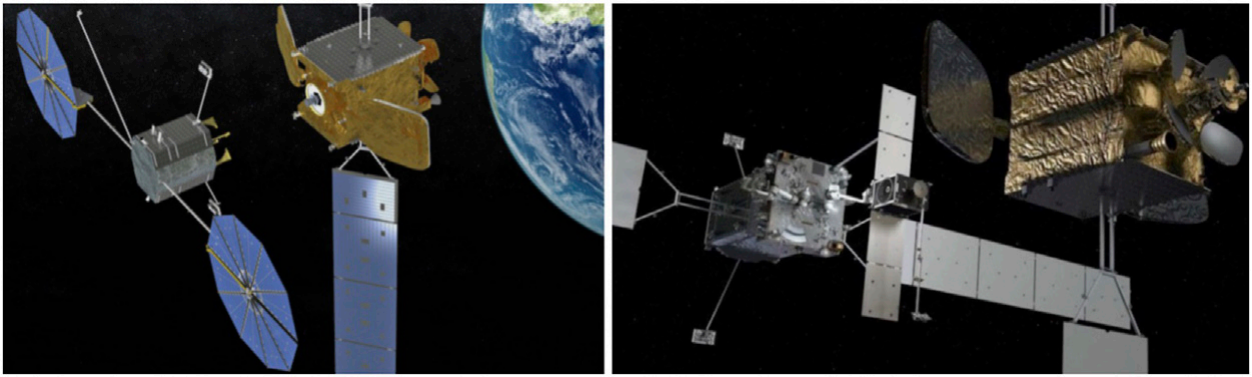
Northrop Grumman’s **(A)** MEV (Orbital ATK), and **(B)** MRV (Grumman, 2021).

Orbital ATK (now part of Northrop Grumman) has developed the Mission Extension Vehicle (MEV) missions in the past few years. They were the first OOS missions developed by a private company purely for commercialization. MEV-1 was launched in October 2019 and completed its historic docking with the Intelsat 901 spacecraft on February 25, 2020. This marked the very first time two commercial satellites docked in orbit. IS-901 resumed communications services on April 2, 2020. MEV-2 was launched and successfully docked with the Intelsat 10-02 (IS-10-02) on April 12, 2021 (Grumman, 2021). MEV-2 is the second Mission Extension Vehicle supplied by Space Logistics LLC, a wholly owned subsidiary of Northrop Grumman. Unlike MEV-1, which docked client IS-901 above the GEO orbit before moving it back into service, MEV-2 docked with the client IS-10-02 directly in its operational GEO orbital location. Both MEV-1 and MEV-2, shown in Figure 10A, are planned to extend their client satellites for a five-year period after which the clients will be placed back into graveyard orbit. Then the servicing vehicles will have an option to service other client satellites on orbit.

Northrop Grumman has recently teamed with DARPA with the RSGS program for the new Mission Robotic Vehicle (MRV), as shown Figure 10B. In the MRV mission, DARPA will provide the robotics payload that will be used to service satellites at a GEO orbit.

The United States Naval Research Laboratory developed the payload for the RSGS program. It consists of two dexterous robotic arms, along with several tools and sensors. Northrop Grumman’ Space Logistics division will provide the bus technologies it developed for the MRV mission (Erwin, 2020).

### Other General Software and Hardware Technology Developments

The mission activities go hand in hand with technology developments. In the COMRADE project, ESA has promoted the design, development, and testing of a control system for a free-flying robot for two missions: a) Active Debris Removal (ADR) with a dedicated seven DoF robotic manipulator and LAR gripper end-effector. b) Refueling mission (see also *Arms, Grippers, and End-Effectors*) Here, a combined controller was tested on the OOS-SIM facility for the capturing of ENVISAT. The controller ran on a LEON4 computer, proving its applicability for space flight. Furthermore, an overview of the design and outcomes of the project were presented in (Colmenarejo et al., 2018), to include a comparison between a robust $H_{\infty }$ controller and a nonlinear Lyapunov-based controller. The results from Monte Carlo simulations showed that although the $H_{\infty }$ controller performed better in meeting the given velocity requirements, the nonlinear controller was usually able to achieve a stable and successful grasp in presence of contact. The nonlinear controller was also presented in detail in De Stefano et al. (2021), including results from experiments performed on DLR’s OOS-SIM experimental facility.

Other important software and hardware developments have been undertaken in Europe under the six-year PERASPERA project (PERASPERA, 2014), within the EU Strategic Research Cluster on Space Robotics, aiming, among other things, at the maturation of orbital robotic technologies. The first set of grants (Operational Grants) within this project (2016–2017) was dedicated to the development of common building blocks, to include an operating system or middleware (European Space Robot Control Operating System, ESROCOS), a planning framework (European Robotic Goal-Oriented Autonomous Controller, ERGO), a sensor data fusion framework (InFuse), an integrated sensor suit (I3DS) and a standard interface for robotic manipulation of payloads (SIROM).

Running at the DLR since 2014, the RICADOS project aims at holistic simulation of an on-orbiting servicing mission, from a realistic ground segment (GSOC) to a communication link to a space segment, performing inspection, rendezvous, and capture tasks (Benninghoff et al., 2018). These tasks are partly validated on DLR’s hardware-in-the-loop facilities EPOS and OOS-SIM. Furthermore, the DLR, in cooperation with the MIT, is promoter and developer of an experimental mission with the ASTROBEEs on the ISS, for the approach maneuver of a chaser satellite to a tumbling target. In this mission, the functional sequence consisting of motion prediction, motion planning and robust trajectory tracking, will be tested (Albee et al., 2021). The telepresence technology has also been extensively demonstrated by the DLR in different projects, to include KONTUR-2 (Artigas et al., 2016; Riecke et al., 2016) and METERON (Schmaus et al., 2018).

The initial PERASPERA building blocks were then used in a second set of grants, which developed concepts and technologies for a servicing mission (EROSS), for modular spacecraft assembly and reconfiguration (MOSAR) and for on-orbit assembly of a large space telescope (PULSAR). The goal is to perform an orbital demonstration mission in 2023–24.

**FIGURE 11:**
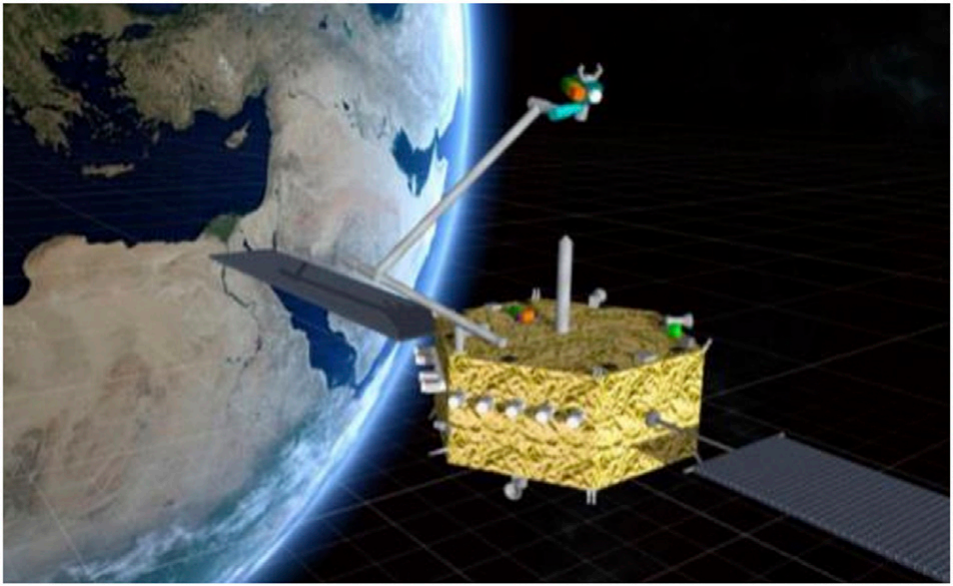
The EROSS Concept includes a free-flying spacecraft equipped with a 7-DoF manipulator (EROSS EU Horizon 2020).

In particular, the EROSS project assesses and demonstrates the capability of a manipulator-equipped servicing spacecraft to perform medium and close-range rendezvous, and then to capture and manipulate/service a collaborative client satellite with a highest degree of autonomy, see Figure 11. EROSS reuses and integrates both software (such as ESROCOS, ERGO and INFUSE) and hardware (such as SIROM), developed in previous Operational Grants led in PERASPERA, and previous developments on projects by the European Space Agency, such as the ASSIST project. When required, new designs are produced, such as a new manipulator gripper. To facilitate the varying demands of the different stages of the mission, a versatile GNC architecture is developed, including a Coordinated Control scheme that allows for the simultaneous Model-based PD control of the servicer platform attitude and Compliant Control of the end-effector of the seven Dof manipulator (Dubanchet, et al., 2020).

### Arms, Grippers, and End-Effectors

**FIGURE 12:**
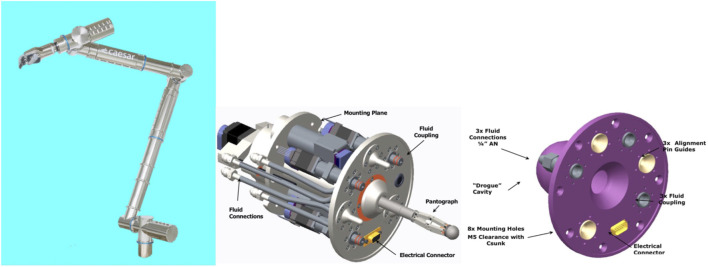
**(A)** CAESAR robot arm with SpaceHand (DLR), **(B)** ASSIST mechanism for refueling satellites (ESA).

The development of hardware for orbital robotics has been very active in the last years. The DLR had tested and validated its robot joint technology in the ROKVISS mission (Yoshida et al., 2016), in which two robot joints were placed on the outer surface of the ISS, between 2005 and 2011. In a recent development, this technology has been improved and used to build a seven-degree of freedom robot manipulator, the Compliant Assistance and Exploration SpAce Robot (CAESAR), shown in Figure 12A (Beyer et al., 2018). Other robotic arms are being developed in the United States, to include DARPA’s FREND arm, as well as the Dragonfly, later developed into the longer SPIDER. A torque-controlled robot is also constructed by TUI with the name of KRAKEN.

ESA focused on developing a cost-effective solution for refueling GEO satellites in space as currently the fuel levels often deplete, for communications satellites, while the payloads are still in good health. A refueling mechanism was developed, called ASSIST, which will allow satellites in the future to be refueled and serviced while on orbit, extending their life, Figure 12B. As is typically the case for most end-effectors, the ASSIST mechanism performs first *soft* docking (allowing relative motions but not separation) followed by a motorized retraction ending during a *hard* docking phase (rigidization) using aligning pins (Medina et al., 2017). ASSIST is the reference mission with dedicated 6/7 DoF robotic manipulator and ASSIST end-effector (Visentin, 2020).

In ESA project Predator, the main objective was to design and prototype/demonstrate via functional tests a robotic end-effector gripper breadboard that can be used to capture the Launch Adaptor Ring (LAR) of non-cooperative satellites during a debris removal mission (Visentin, 2020). The Stewart platform-based gripper, which is attached to the tip of the robotic arm, plays an important role in the satellite capture operation as it provides the mechanical and structural interface between the servicer/chaser vehicle and the target satellite during the critical capture and stabilization operations.

Other robot end-effector designs can be found in Jaworski et al. (2017) and Jaekel et al. (2018) for a mechanism which can also clamp to the launch adapter ring of the target satellite and in Trentlage et al. (2016) and Cauligi et al. (2020) for Gecko-inspired grippers. A tool for capturing a non-cooperative target is described in Sun et al. (2020).

#### Chinese Space Station Manipulators

**FIGURE 13:**
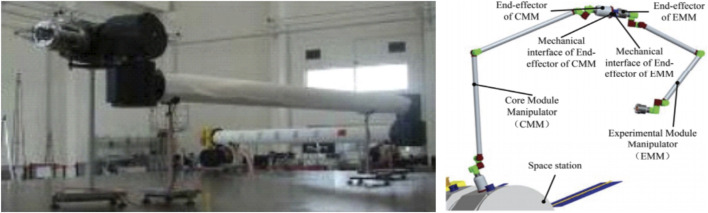
**(A)** Chinese Space Station Manipulator (CSSM) (Li et al., 2015), **(B)** China’s Space Station Remote Manipulator System (CSSRMS) (Liu, 2014).

The China’s Space Station Remote Manipulator System (CSSRMS) consists of two manipulators: a larger manipulator called Chinese Space Station Manipulator (CSSM) and a smaller one called Experimental Module Manipulator (EMM) (Li et al., 2019). The CSSM is a 10.5-m long, 7-joint manipulator designed for transposing and assembling large station modules, handling transportation cargos, assisting the smaller manipulator EMM or an astronaut as a mobile platform (Li et al., 2015). It can manipulate a large payload of 25,000-kg mass at maximum tip linear and angular velocities of 0.02-m/s and 0.15-°/s, respectively, while its unloaded tip velocities can reach up to 0.3-m/s and 3-°/s, respectively, Figure 13A.

With its symmetric topology design of two identical ends (Figure 13B), it can relocate itself on the station using its “walking” capability. EMM is a 5-m long, 7-joint manipulator designed for taking care of the station’s exposed experimental platform and optical platform and providing support to EVA activities (Liu, 2014). It can handle a payload of 3,000-kg mass at maximum tip linear and angular speeds of 0.03-m/s and 0.15-°/s, respectively, while its unloaded tip speeds can reach up to 0.2-m/s and 3-°/s. It can operate either from an anchor point on the station or from the tip of CSSM which extends its tip reachability to over 15 m. Both manipulators have force-motion control capability and can be operated either in automated mode or remotely control mode from the space station or a ground station. The two manipulators will be launched to space in 2021–2022 timeframe (Yang, 2021).

#### Gateway Extravehicular Robotic System

**FIGURE 14:**
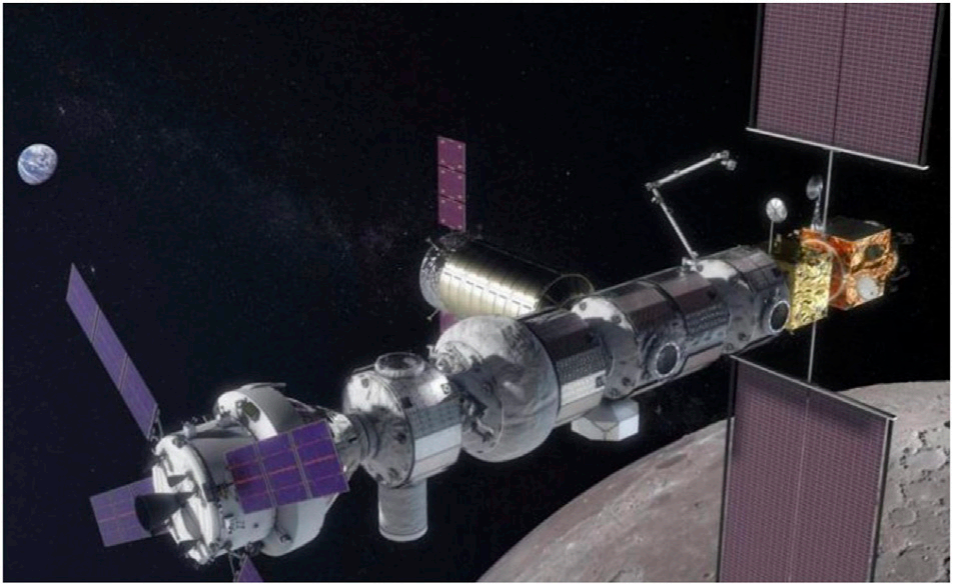
The gateway extravehicular robotic system (GERS).

The Lunar Gateway is considered by NASA and the ISS partners for the next flagship human space exploration. The International Space Exploration Coordination Group (ISECG) has also concluded that the Gateway will be critical in expanding human presence to the Moon, Mars and deeper into the Solar System (Merrill et al., 2015; Shireman et al., 2018). The Gateway is a crewed orbiting platform like the ISS, but instead of operating in Low Earth Orbit (LEO), the Gateway will operate in a Moon-centric orbit called near-rectilinear halo orbit (NRHO). Canada contribution to the Gateway program, as a partner of the ISS, is intended to be an extra-vehicular robotics (EVR) system, which is deemed to be the evolution of the iconic robotics element of the ISS known as the Canadarm 2; see Figure 14. The Gateway EVR system as shown in Figure 14 is intended to provide similar services to the Gateway as the Canadarm 2 provides for the ISS, such as maintenance, remove and replacement (R&R) operation of ORUs, inspection, as well as berthing of commercial and international cargo spacecraft to visit Gateway regularly for bringing fresh supply. However, there are new services proposed. One interesting application of the EVR system proposed on the Gateway is the assisted attitude control system (ACS), which can lead to significant fuel saving (Aghili and Rey, 2020).

Traversal of the lunar Gateway along its designated NRHO orbit can impart significant angular momentum to the spacecraft from lunar gravity gradient torques. The stored angular momentum cannot be removed by a magnetic torquer since the magnetic field of the Moon is very weak, while using thrusters may require propellant consumption of 9 kg/year. A cost-effective solution based on a robotically steerable solar sail permitting desaturation of the Gateway’s wheels without using any propellant is proposed in Aghili and Rey (2020). The solar sail is grasped and then optimally positioned and oriented by the designated robotic arm of the Gateway to generate the required torque through solar radiation pressure.

### Reconfigurable Robots for On-Orbit Servicing

Robotic manipulators working in space environments often need to change their configuration to meet the demands of a specific task within the constraints of the environment. Particularly in space applications, it is desirable and cost-effective to employ a single versatile robot capable of performing different tasks such as inspection, contact operation, assembly (insertion and removal of objects), and carrying objects (pick and place). Optimal operation of each of these tasks demands a specific manipulator design. For instance, large robots maximizing the structural length index are typically suitable for inspection, robots with maximum manipulability measure are well conditioned for dexterous contact tasks, and configurations maximizing the distance of the robot limbs and extremities from the environment are suitable for payload handling.

**FIGURE 15:**
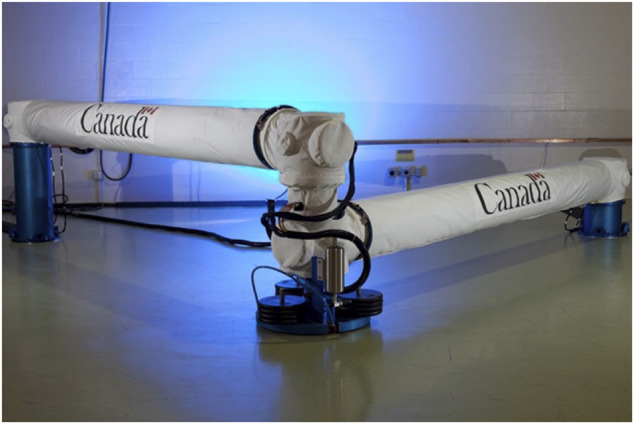
The next-generation Canadarm based on the telescopic link reconfiguration concept (CSA).

Space systems are designed for minimum weight to reduce launch cost. Another design constraint for a space system is that it should be compact enough to be accommodated within its designated space in the launch vehicle. Since the links of a space manipulator are usually long, they must be folded before launch. For example, the Canadarm 2 has two long booms, each of which has a hinge at the middle, which allowed the booms to be folded before launch and then unfolded manually by astronauts in orbit. For on-orbit servicing missions whereby no human operator is present, the robot must be able to deploy itself. The original reconfigurable robot was introduced in Fukuta and Kawauchi (1990) to add versatility to the robotic manipulator. The concept was then developed further in Paredis and Khosla (1995) Cellular robots based on hexagonal modules and the concept of robot molecules was described in Kotay et al. (1998). Another modular reconfigurable manipulator, which had three 6-degree-of-freedom (DoF) arms, was proposed for space applications in Ohkami et al. (1999), Hayashi et al. (2000) and Shibata and Ohkami (2002); the manipulator was designed to be able to brachiate around the Japanese section of the ISS. All these reconfigurable robots are modular, hence needing an effective docking system for connecting and releasing the modules. Reconfigurable robots based on modular joints have been proposed for both terrestrial and space applications (Aghili and Su, 2012). Although modular robots have the great advantage of being able to change their number of links and to create a tree-like structure, they require complex joints for connecting modules, as well as a docking system for exchanging modules. Reconfigurable robots based on lockable telescopic links was first proposed in Aghili and Parsa (2009) to offer a simpler and more effective solution to the problem. Further to the versatility that this design provides, it makes it possible to contain the manipulator in a small volume, which is suitable for launch, see Figure 15.

## Conclusion

This survey addressed fundamental aspects of manipulation and capture on orbit, such as the dynamics of SMSs in orbit, the contact dynamics between manipulator grippers and their targets, and the methods for identifying properties of SMSs and of their targets. Also, it presented recent work in the areas of sensing of pose and system states, of motion planning for capturing a target, and of feedback control methods for SMS to perform challenging motion or interaction tasks on orbit. Finally, the paper reviews major ground-based test and verification facilities developed by space agencies across the world for on-orbit robotic capture and service operations, and several recent or near-future missions and technologies developed for technology demonstration and commercialization. Although a lot of research work has been done recently, several important issues remain open and need to be studied, enabling safe and successful proliferation of robotic systems in orbit. The survey discussed these remaining challenges and issues.
